# *Androglobin* gene expression patterns and FOXJ1-dependent regulation indicate its functional association with ciliogenesis

**DOI:** 10.1016/j.jbc.2021.100291

**Published:** 2021-01-13

**Authors:** Teng Wei Koay, Carina Osterhof, Ilaria M.C. Orlando, Anna Keppner, Daniel Andre, Schayan Yousefian, María Suárez Alonso, Miguel Correia, Robert Markworth, Johannes Schödel, Thomas Hankeln, David Hoogewijs

**Affiliations:** 1 Section of Medicine, Department of Endocrinology, Metabolism and Cardiovascular System, University of Fribourg, Fribourg, Switzerland; 2 Faculty of Biology, Institute of Organismic and Molecular Evolution, Molecular Genetics & Genome Analysis, Johannes Gutenberg University Mainz, Mainz, Germany; 3 Institute of Physiology, University of Duisburg-Essen, Duisburg, Germany; 4 Department of Nephrology and Hypertension, Universitätsklinikum Erlangen and Friedrich-Alexander-Universität Erlangen-Nürnberg, Erlangen, Germany

**Keywords:** gene expression, transcription factor, transcription regulation, transcription enhancer, transcriptomics, hemoglobin myoglobin, cilia, CRISPR/cas, bioinformatics, ADGB, androglobin, ALI, air–liquid interface, ANOVA, analysis of variance, ChIP, chromatin immunoprecipitation, CYGB, cytoglobin, HB, hemoglobin, MB, myoglobin, mTEC, mouse tracheal epithelial cells, NGB, neuroglobin, PFA, para-formaldehyde, RT, reverse transcription, sgRNA, single guide RNA, TSS, transcriptional start site

## Abstract

Androglobin (ADGB) represents the latest addition to the globin superfamily in metazoans. The chimeric protein comprises a calpain domain and a unique circularly permutated globin domain. ADGB expression levels are most abundant in mammalian testis, but its cell-type-specific expression, regulation, and function have remained unexplored. Analyzing bulk and single-cell mRNA-Seq data from mammalian tissues, we found that—in addition to the testes—ADGB is prominently expressed in the female reproductive tract, lungs, and brain, specifically being associated with cell types forming motile cilia. Correlation analysis suggested coregulation of ADGB with FOXJ1, a crucial transcription factor of ciliogenesis. Investigating the transcriptional regulation of the *ADGB* gene, we characterized its promoter using epigenomic datasets, exogenous promoter-dependent luciferase assays, and CRISPR/dCas9-VPR-mediated activation approaches. Reporter gene assays revealed that FOXJ1 indeed substantially enhanced luciferase activity driven by the *ADGB* promoter. ChIP assays confirmed binding of FOXJ1 to the endogenous *ADGB* promoter region. We dissected the minimal sequence required for FOXJ1-dependent regulation and fine mapped the FOXJ1 binding site to two evolutionarily conserved regions within the *ADGB* promoter. FOXJ1 overexpression significantly increased endogenous ADGB mRNA levels in HEK293 and MCF-7 cells. Similar results were observed upon RFX2 overexpression, another key transcription factor in ciliogenesis. The complex transcriptional regulation of the *ADGB* locus was illustrated by identifying a distal enhancer, responsible for synergistic regulation by RFX2 and FOXJ1. Finally, cell culture studies indicated an ADGB-dependent increase in the number of ciliated cells upon overexpression of the full-length protein, confirming a ciliogenesis-associated role of ADGB in mammals.

Globins are small globular metallo-proteins consisting of about 150 amino acids, which comprise eight α-helical segments in a characteristic 3-over-3 α-helical sandwich structure. This conserved “globin fold” identifies them as members of a large protein superfamily. Globins contain a heme prosthetic group, by which they can reversibly bind gaseous ligands such as O_2_, CO, and NO. Historically, the familiar vertebrate O_2_-binding hemoglobin (HB), a tetramer of α- and β-globins, and the monomeric myoglobin (MB) were among the first proteins whose sequences and structures were determined already over 50 years ago. Genomic analyses have considerably altered and extended our view of the globin family in mammals, leading to the discovery of novel globin types such as neuroglobin (NGB) and cytoglobin (CYGB), which are expressed in nerve and fibroblast-like cells, respectively (1, 2). Both globin types perform yet-to-be-illuminated functions, which possibly reside in antioxidant defense, reactive oxygen species signaling, or even lipid metabolism (3, 4).

Recently, a novel family of large, chimeric proteins containing a globin-like domain was discovered and termed androglobin (ADGB) based on its preferential expression in mammalian testis tissue (5). ADGB is a chimeric protein of about 1500 amino acids, which contains an embedded globin domain. This globin domain is permutated with respect to its characteristic alpha helices and interrupted by a calmodulin-binding motif. Nevertheless, the globin domain appears to be able to bind oxygen *in vitro* (5). The N-terminal domain of ADGB shows high sequence similarity to the human protease calpain 7, although functionally important amino acid residues are mutated. ADGB was shown to be highly conserved throughout the metazoan tree of life, and orthologous copies of the *ADGB* gene could be found from humans and other vertebrates down to very basal taxa such as the cnidarian *Nematostella vectensis*, the placozoan *Trichoplax adherens*, and even the choanoflagellate *Monosiga brevicollis* (5), which suggests an elementary and possibly conserved function in metazoans. ADGB is predominantly expressed in later stages of spermatogenesis in mammalian testes and, to a much lower extent, in the lung and brain tissue (5). An important role of ADGB in spermatogenesis was supported by analysis of published microarray data revealing that endogenous levels of human ADGB mRNA were lower in the testes of infertile men *versus* their healthy counterparts. An *in vitro* cell culture study suggested that ADGB could act as an oncogene in glioma formation and an ADGB knockdown could inhibit the growth of glioma cell lines (6). Overall, studies on ADGB expression patterns and gene regulation were scarce, and the functional role of ADGB has remained elusive.

Since the expressional profile of a gene, specifically addressing the organs and their cell types, can provide a valuable hint at its possible function (as illustrated *e.g.*, by the specific presence of HB in erythrocytes), we revisited the expression patterns of ADGB using an integrative approach of bioinformatical data mining. In particular, novel RNA-Seq datasets from bulk and single-cell experiments were analyzed with the aim to recognize common patterns with functional implications. The data yielded valuable insight into the properties of ADGB-expressing cell types, which led us to characterize in detail the gene-regulatory landscape determining ADGB expression. We comprehensively mined epigenomic databases for accessible chromatin and promoter/enhancer-associated histone marks, identified transcription factors binding to the *ADGB* locus using reporter gene assays and chromatin immunoprecipitation (ChIP) experiments, and further characterized several functional distal enhancers in the *ADGB* locus. Finally, we performed ADGB overexpression *in vitro* to elicit a cellular phenotype. These different lines of experimental evidence converged and convincingly pointed out that the cellular function of ADGB is associated with the presence of motile cilia.

## Results

### ADGB expression in female reproductive tract, lung, and brain suggests functional association with ciliary structures

**Figure 1:**
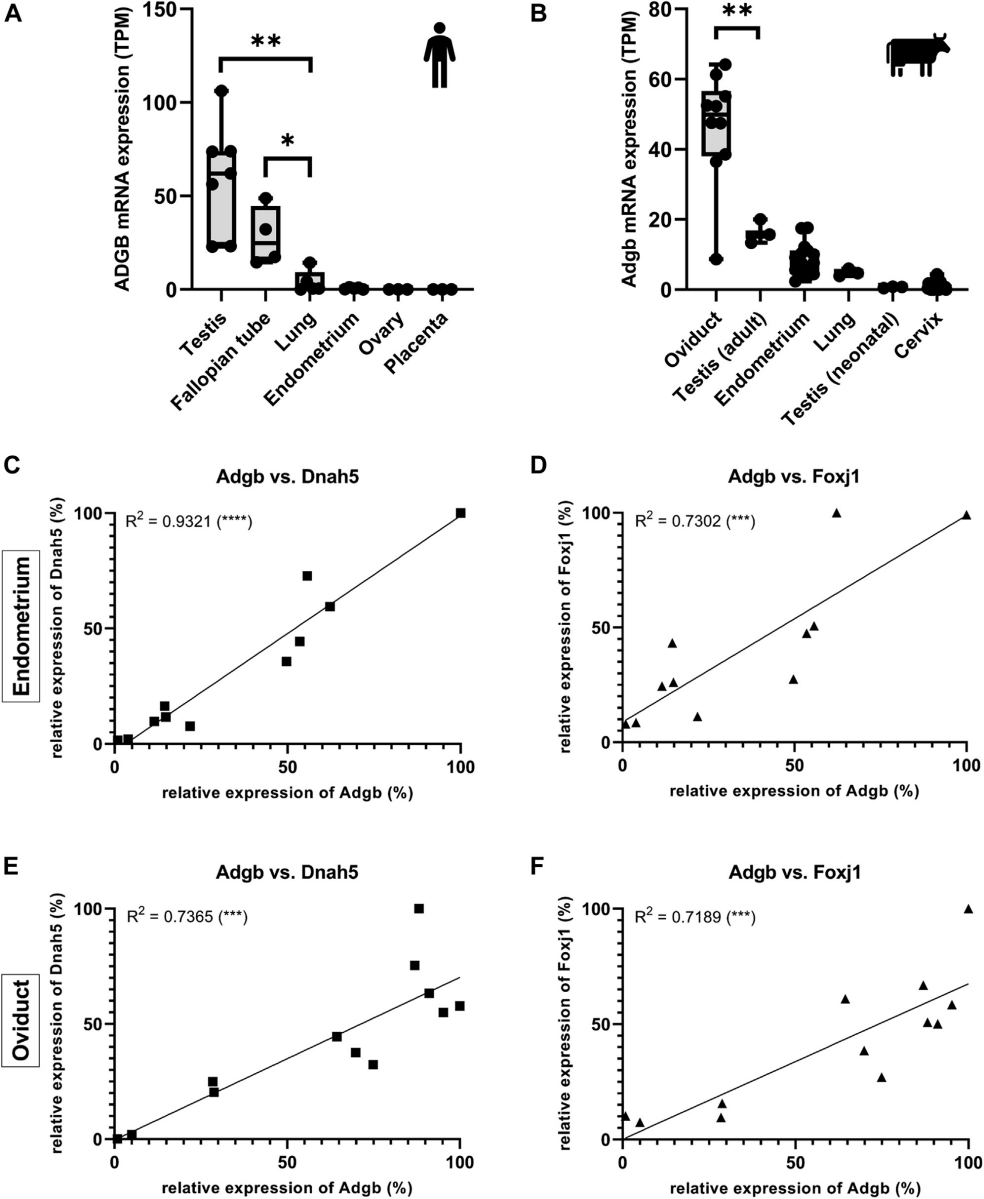
**Novel Adgb expression sites correlate with cilia-associated genes.***A* and *B*, expression levels of Adgb mRNA in human and bovine tissues as determined by bulk RNA-Seq. *A*, high levels of expression are found in human testis, but also in fallopian tubes of females. *B*, transcript levels of Adgb in the oviducts of cattle exceed expression in bovine testis. *C*–*F*, correlation analysis of Adgb mRNA expression and mRNA levels of cilia-associated genes Dnah5 (*left*) and Foxj1 (*right*). Expression was measured by RT-qPCR analysis in the endometrium (n = 11, *upper panels*) and oviduct (n = 12, *lower panels*) of cattle. Adgb shows very strong correlation with Dnah5 (R^2^ = 0.93) in the endometria (*A*), strong correlation (R^2^ = 0.73) with Dnah5 in the oviducts (*C*), and strong correlations with Foxj1 in the endometria (R^2^ = 0.73, B) as well as oviducts (R^2^ = 0.72, D). ∗*p* < 0.05; ∗∗*p* < 0.01; ∗∗∗*p* < 0.001; ∗∗∗∗*p* < 0.0001.

The wealth of gene expression data, which have been produced since the initial description of ADGB in 2012, enabled us to define a much more detailed expression profile of the gene in mammalian tissues and cell types. As such, the bulk RNA-Seq data of the Human Protein Atlas (7) revealed the fallopian tube of the female reproductive tract as a novel expression site of ADGB mRNA (Fig. 1*A*). Transcript levels were even higher than in the lung, which was initially described as the second highest ADGB-expressing human organ (5). To study this further, and noticing a shortage of data from healthy human samples, we evaluated bulk RNA-Seq data from the female reproductive tract of cattle. The bovine data sets confirmed Adgb expression in the oviducts, showing the highest amount of Adgb expression of all cattle organs analyzed, and in endometrial tissue (Fig. 1*B*). Human endometrial data appeared largely devoid of ADGB RNA, but sequencing data of separate stromal and epithelial fractions (8) revealed restriction of ADGB expression to the epithelial fraction only (Fig. S1).

Both human and cattle RNA-Seq data revealed a high interindividual variability in expression intensity, which suggested a temporal and/or spatial restriction of Adgb expression in the female reproductive tract. To study the gene expression footprint of the hypothetical Adgb-expressing cell type involved, we subdivided the bovine endometrial samples into 2 groups, “Adgb-high” (TPM >20, n = 4) and “Adgb-low” (TPM <5, n = 4), and performed differential gene expression analysis to infer genes associated with either high or low levels of Adgb. Subsequent overrepresentation analysis (Table 1, Supplemental File 1) revealed that genes associated with high amounts of Adgb were connected to GO-terms such as “cilium and axoneme assembly”, “dynein-dependent microtubular transport”, “microtubular movement” and, interestingly, the “sperm flagellum.” An independent clustering approach to identify genes with an Adgb-type expression pattern using an additional data set of human fallopian tube samples (9) generated a smaller subset of genes, which were even more strongly associated with cilia-related processes such as “cilium movement”, “determination of left-right-symmetry” and the “differentiation of lung epithelial cells” (Table 2, Supplemental File 2). Among these approximately 100 Adgb-associated genes, we found Foxj1, the master transcription factor of ciliogenesis (10), and Dnah5, a protein known for its specific localization to motile cilia of the respiratory tract (11). The tissue with the highest amount of Adgb expression, however, was inconsistent between the samples of the two species (Fig. 1, *A* and *B*). A possible explanation could be that the samples were at different stages of the menstrual cycle, given that ciliogenesis is estrogen-dependent (12, 13). Additionally, depending on the part of the oviduct that was dissected, the ratio of epithelial cells to connective tissue and thus the overall number of ciliated cells may vary between samples (14). Reproductive aging (*i.e.*, menopause), which decreases the number of ciliated cells (15), may also have contributed to the observed Adgb expression differences.

**Table 1:** GO term enrichment analysis of genes coregulated with Adgb in differential gene expression analysis of bovine endometrial samples

Gene set	Description	Enrichment ratio	FDR
Biological process			
*GO:0000226*	Microtubule cytoskeleton organization	8.37	0
*GO:0060271*	Cilium assembly	17.66	0
*GO:0007018*	Microtubule-based movement	16.96	0
*GO:0035082*	Axoneme assembly	52.00	0
*GO:0003341*	Cilium movement	45.50	0
*GO:0070286*	Axonemal dynein complex assembly	58.50	0
*GO:0007368*	Determination of left/right symmetry	19.31	2.56E-09
*GO:0030317*	Flagellated sperm motility	15.36	6.96E-05
*GO:0001539*	Cilium or flagellum-dependent cell motility	29.25	1.11E-04
*GO:0042073*	Intraciliary transport	24.38	2.97E-04
*GO:0044458*	Motile cilium assembly	27.00	0.0022
Cellular component			
*GO:0015630*	Microtubule cytoskeleton	6.16	0
*GO:0005929*	Cilium	14.60	0
*GO:0031514*	Motile cilium	27.16	0
*GO:0005930*	Axoneme	43.15	0
*GO:0036126*	Sperm flagellum	26.81	0
*GO:0097729*	9+2 Motile cilium	25.74	6.18E-15
*GO:0097223*	Sperm part	17.10	3.49E-14
*GO:0005815*	Microtubule organizing center	6.19	5.86E-12
*GO:0044447*	Axoneme part	56.57	8.32E-12
*GO:0005858*	Axonemal dynein complex	68.95	1.69E-09
*GO:0005875*	Microtubule-associated complex	14.45	7.82E-09
Molecular function			
*GO:0003774*	Motor activity	15.80	5.35E-06
*GO:0003777*	Microtubule motor activity	21.23	1.81E-05
*GO:0015631*	Tubulin binding	7.11	4.41E-05
*GO:1990939*	ATP-dependent microtubule motor activity	30.79	0.0021
*GO:0008017*	Microtubule binding	6.73	0.0061
*GO:0008092*	Cytoskeletal protein binding	3.23	0.0087
*GO:0045504*	Dynein heavy chain binding	37.53	0.0087
*GO:0045503*	Dynein light chain binding	37.53	0.0087

All GO terms show a strong connection to the motile cilium. The associated gene list and the full list of enriched terms are provided in Supplemental File 1.

**Table 2:** Enriched GO terms in genes showing correlation with ADGB expression in human fallopian tube samples

Gene set	Description	Enrichment ratio	FDR
Biological process			
*GO:0007017*	Microtubule-based process	6.02	1.28E-04
*GO:0035082*	Axoneme assembly	28.76	1.96E-04
*GO:0003341*	Cilium movement	28.30	1.96E-04
*GO:0007018*	Microtubule-based movement	9.71	5.76E-04
*GO:0001578*	Microtubule bundle formation	19.71	8.64E-04
*GO:0060271*	Cilium assembly	6.70	0.0088
*GO:0000226*	Microtubule cytoskeleton organization	5.41	0.0146
*GO:0007368*	Determination of left/right symmetry	12.71	0.0439
*GO:0060487*	Lung epithelial cell differentiation	41.77	0.0439
*GO:0009855*	Determination of bilateral symmetry	11.89	0.0452
Cellular component			
*GO:0005929*	Cilium	12.87	0
*GO:0005930*	Axoneme	30.26	3.82E-10
*GO:0097014*	Ciliary plasm	30	3.82E-10
*GO:0031514*	Motile cilium	19.38	2.28E-08
*GO:0005858*	Axonemal dynein complex	76.67	2.73E-05
*GO:0005875*	Microtubule-associated complex	16.32	2.86E-05
*GO:0005874*	Microtubule	6.01	0.0109
*GO:0097729*	9+2 Motile cilium	14.68	0.0109
*GO:0036157*	Outer dynein arm	76.67	0.0191
*GO:0070160*	Tight junction	10.95	0.0284
Molecular function			
*GO:1990939*	ATP-dependent microtubule motor activity	45.47	6.49E-06
*GO:0045503*	Dynein light chain binding	66.68	9.42E-06
*GO:0003777*	Microtubule motor activity	24.10	1.06E-04
*GO:0003774*	Motor activity	14.82	0.0014
*GO:0051959*	Dynein light intermediate chain binding	34.49	0.0228
*GO:0045505*	Dynein intermediate chain binding	33.34	0.0228

All GO terms reveal a strong connection to the motile cilium. The associated gene list and the full list of enriched terms are provided in Supplemental File 2.

Experimentally, we confirmed the fallopian tube and the endometrium as novel expression sites *via* RT-qPCR analysis in cattle. In addition, we also determined the amount of Dnah5 and Foxj1 mRNA in these samples (Fig. 1*C*). Foxj1 transcript levels showed a positive correlation with Adgb expression in the endometria and oviducts (R^2^ = 0.73 and R^2^ = 0.72, respectively). The association between Dnah5 and Adgb in the endometrium was even stronger (R^2^ = 0.93). Though not as prominent, there was also a significant positive correlation between expression of Adgb and Dnah5 in the oviducts (R^2^ = 0.74). Immunohistochemistry analysis further confirmed the localization of Adgb protein in the epithelia in the bovine endometrium and specifically in multiciliated cells in the oviduct (Fig. S2).

**Figure 2:**
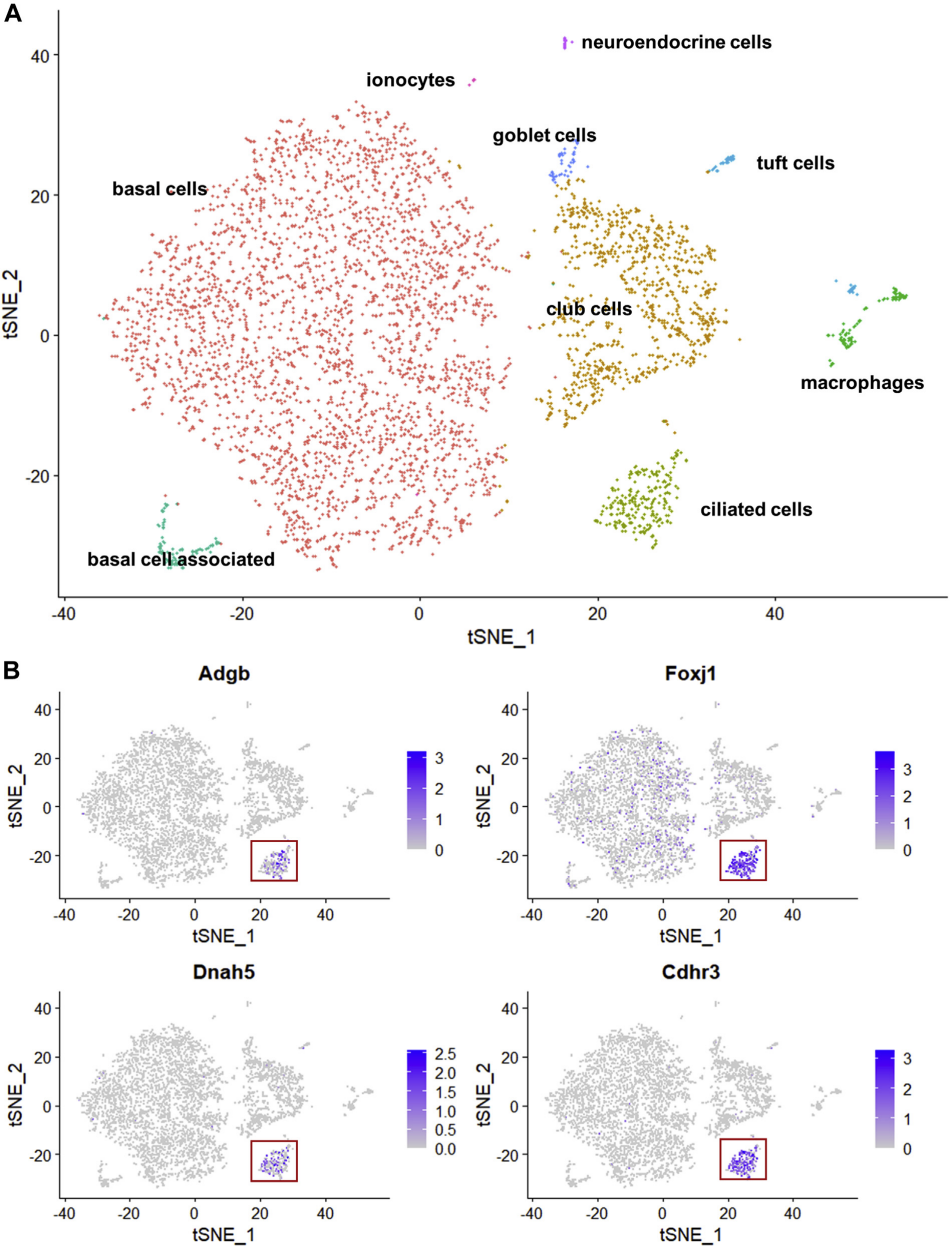
**Clustering analysis of single cell RNA-Seq data from murine lungs.***A*, tSNE representation of cell clusters (named in accordance with (16)). *B*, visualization of all clusters expressing mRNA of Adgb and ciliary marker genes Dnah5, Foxj1 and Cdhr3. Adgb expression is restricted to ciliated cells in murine lung epithelia.

We previously reported the lung to show the second highest Adgb mRNA expression, after the testes (5). Bulk RNA-Seq data from this tissue, however, led to inconclusive results with high interindividual variability and overall low levels of expression, or, as in some human samples, no expression at all (Fig. 1). Therefore, we considered analyzing available single-cell RNA-Seq data obtained from the murine lung (16). To prove that this method was sensitive enough to detect Adgb mRNA expression, we first reanalyzed single-cell RNA-Seq data from murine testis (17). We could show that, in accordance with Hoogewijs *et al.* (5), Adgb mRNA expression was restricted to later stages of spermatogenesis, where round spermatids differentiate into elongating spermatids and form the flagellum, a motile microtubular structure very similar to a motile cilium. Fully differentiated condensed spermatids, however, did not express Adgb mRNA anymore (Fig. S3). Following this proof of principle, we performed clustering analysis on single-cell RNA-Seq data sets from epithelial fractions of murine lungs (dataset from Montoro *et al.* (16)). This revealed a distinct entity of lung cells expressing Adgb. Using known cell-type markers from literature, and in accordance with our original report, we identified these cells as being multiciliated (Fig. 2). As we had observed in the correlation analysis on fallopian tube samples, Adgb expression correlated well with Dnah5 and Foxj1, although the overall number of Adgb-positive cells was lower. An additional round of clustering of these ciliated cells revealed no subtypes with noticeable differences in Adgb expression, so that we assume that Adgb-negative ciliated cells are due to dropout artifacts because of rather low endogenous levels of Adgb mRNA (Fig. S4). No Adgb expression was observed in progenitors of multiciliated cells, such as basal cells (Fig. 2). Cell subcluster 4 (Fig. S4) showed slightly lower levels of both, Adgb and Foxj1, but a higher amount of expression of the basal cell marker Aqp3 (16). This could indicate that Adgb expression rises during differentiation and is rather associated with later stages of ciliogenesis or with a maintenance function in cells with already established cilia.

**Figure 3:**
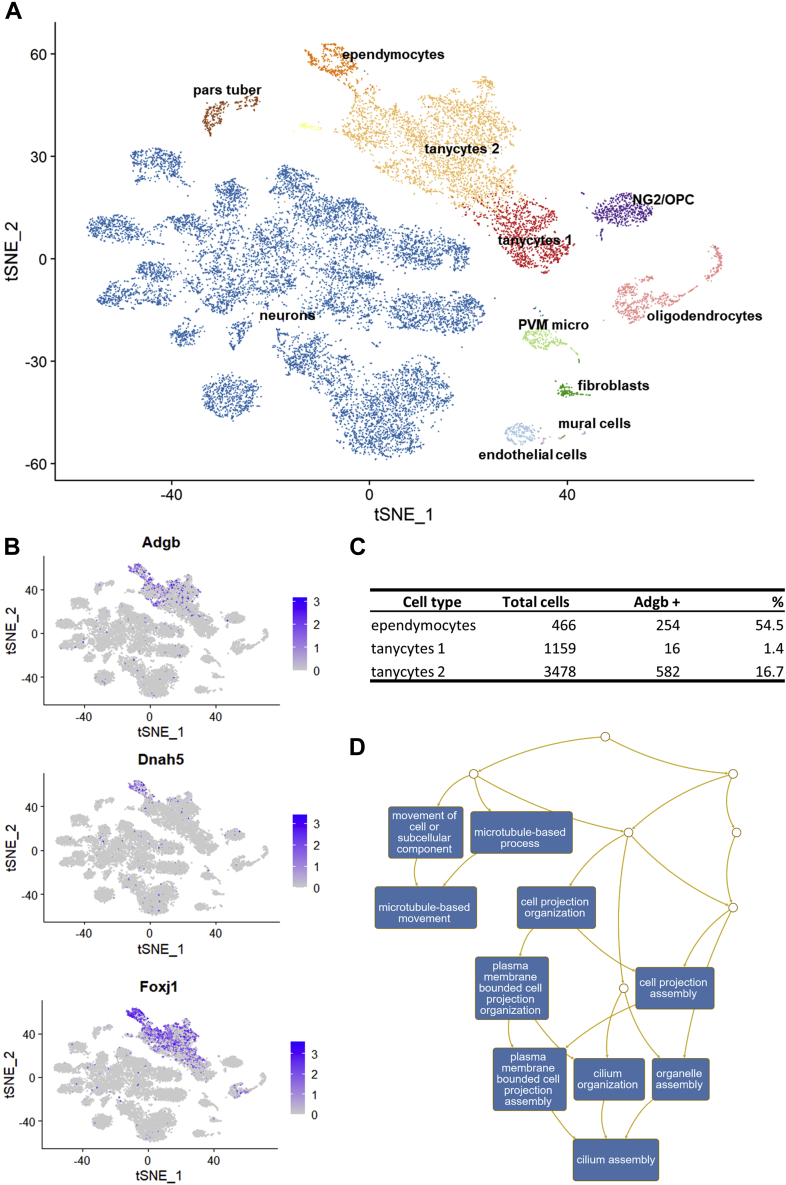
**Analysis of single cell RNA-Seq data from mouse hypothalamus.***A*, tSNE representation of brain cells clustered by levels of expression similarity. Cell types were named in accordance with the initial publication (19). *B*, mRNA expression levels of Adgb, Dnah5 and Foxj1. Adgb expression is most prominent in ependymocytes, but also in subpopulation “2” of tanycytes. Foxj1 expression is also found in these two clusters and absent in tanycytes “1”. Dnah5 expression is restricted to ependymocytes. *C*, percentage of Adgb-positive cells in ependymocytes and tanycytes subtypes. *D*, gene ontology analysis of genes overrepresented in ependymocytes and tanycytes “2”. Adgb positivity correlates with terms connected to cilia.

Cells with multiple motile cilia are not only found in the airways and the reproductive tract, but also in the ventricles of the brain, where they maintain proper circulation of cerebrospinal fluid (reviewed in (18)). To obtain further evidence for a functional association of Adgb and motile cilia, and looking to explain the previously reported low expression in brain tissue (5), we reanalyzed single-cell RNA-Seq data from mouse brains enriched for ependymal cells and their neuronal progenitors (19). As expected, we could specifically detect Adgb mRNA expression in fully mature ependymal cells, although only in a small proportion of cells (Fig. 3). In addition, a subpopulation of tanycytes (designated as “2”) showed a moderate amount of Adgb positive cells. GO term analysis of genes overrepresented in ependymocytes and tanycytes “2” again showed a high amount of cilia-associated genes (Fig. 3*D*). Further analysis revealed that Adgb-positive tanycytes belong to the α-subtype, whereas β-tanycytes were Adgb-negative. Although not multiciliated such as lung epithelial cells or ependymocytes, α-tanycytes can be biciliated with the motile 9 + 2 microtubule conformation, whereas β-tanycytes only form 9 + 0 immotile cilia, if any (20). Altogether, these data point at an association of Adgb with cilia formation and/or function and a possible regulation by Foxj1.

### The upstream sequence of the *ADGB* gene displays promoter activity and is inducible by CRISPRa

Gene expression is determined to a great extent by epigenetics and regulatory elements at promoters. As information on this for *ADGB* is scarce, we first inspected data derived from the ENCODE consortium. ENCODE data illustrate that the upstream region surrounding the *ADGB* first exon displays strong DNase hypersensitivity, enrichment of the promoter histone mark H3K4me3, and substantial transcription factor occupancy, all indicating chromatin accessibility and suggesting putative promoter activity (Fig. S5). Furthermore, chromatin segmentation states coupled to HMM motifs suggest promoter activity of this region in six different cell lines. Additional analysis of epigenetic modifications typical of active chromatin regions showed that H3K4me3 was also enriched at this region in multiple additional cell lines. (Fig. S5). This epigenetic profile reflecting open chromatin is in striking contrast to the rather limited, cell-type-specific expression of *ADGB* (see Discussion).

**Figure 4:**
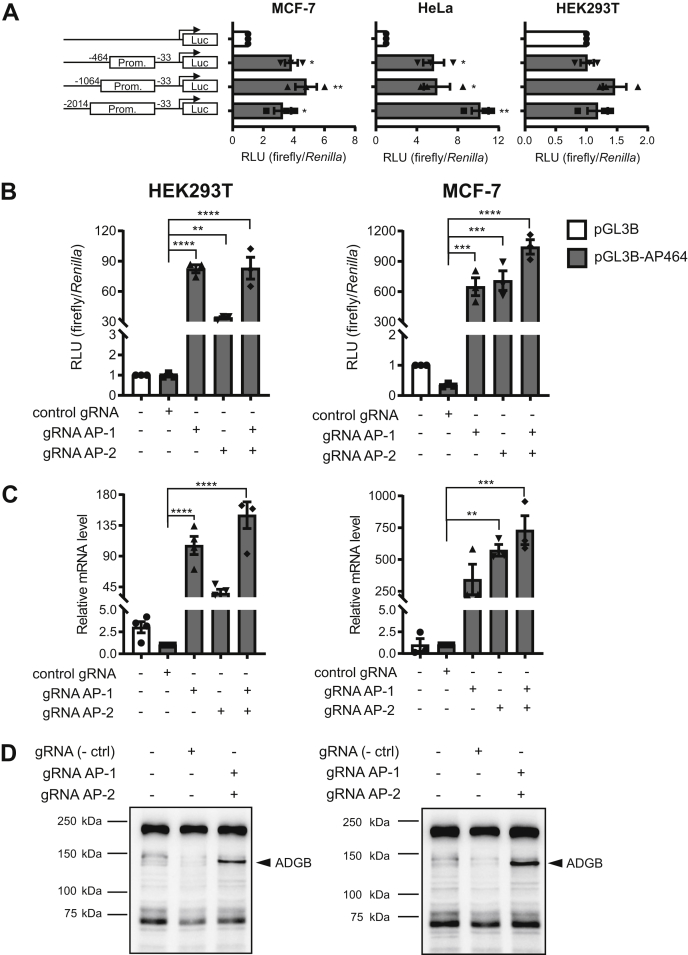
**The upstream region of the *ADGB* transcriptional start site contains promoter activity and is inducible by CRISPRa.***A*, luciferase reporter assays of *ADGB* promoter (AP) elements of three different lengths from −33 bp to −2014 bp, −1064 bp, or −464 bp, respectively, upstream of the *ADGB* TSS in MCF-7 cells, HeLa cells, and HEK293T cells, showing consistent increase in *ADGB* promoter-driven luciferase activity in MCF-7 and HeLa (n = 3 independent experiments). Results are displayed as ratios of firefly to *Renilla* luciferase activities in relative light units (RLU) and normalized to results from pGL3-Basic control transfected cells. Schematic representation of cloned fragments upstream of the *ADGB* gene in a pGL3-Basic vector is shown with numbers representing positions corresponding to the first nucleotide of the TSS. *B*, HEK293T and MCF-7 cells were transfected with dCas9-VPR along with *ADGB* promoter (AP)-targeting gRNAs (gRNA AP-1 and/or gRNA AP-2) and *ADGB* promoter (pGL3B-AP464)-driven luciferase constructs. The gRNA used as negative control contains a nonspecific sequence as present in the pSPgRNA plasmid. Cas9-VPR-based activation of *ADGB* promoter (pGL3B-AP464)-driven luciferase constructs results in activation of the *ADGB* promoter construct (n = 3 independent experiments). Results are displayed as ratios of firefly to *Renilla* luciferase activities in RLU. *C*, HEK293T and MCF-7 cells were transfected with dCas9-VPR along with *ADGB* promoter-targeting gRNA AP-1 and/or gRNA AP-2, and relative *ADGB* transcript levels were quantified by RT-qPCR using a negative control gRNA as reference. Single-guide activation of the *ADGB* promoter with gRNA AP-1 and gRNA AP-2 results in substantial increment in *ADGB* transcript levels (n = 4 independent experiments). Simultaneous expression of gRNA AP-1 and gRNA AP-2 leads to synergistic activation of endogenous ADGB expression (n = 4 independent experiments). *D*, immunoblotting of immunoprecipitated ADGB from HEK293T and MCF-7 cells after gRNAs-dCas9-VPR-activation for 72 h detects endogenous ADGB expression. Data represent mean ± S.E.M (error bars); ∗*p* < 0.05; ∗∗*p* < 0.01; ∗∗∗*p* < 0.001; ∗∗∗∗*p* < 0.0001.

To experimentally explore the basal activity of the putative human *ADGB* promoter, several potential promoter fragments (431 bp, 1031 bp, and 1981 bp long and starting at −33 bp upstream of the transcriptional start site—TSS) were cloned in a pGL3-luciferase basic vector (Fig. 4*A*). Reporter gene assays were performed in three cell lines able to form cilia (21, 22, 23) and displaying reasonable mRNA expression levels of FOXJ1 and RFX2. Following transfection in HeLa and MCF-7 cells, moderate but consistent basal promoter activity could be observed (Fig. 4*A*). No substantial changes were seen in HEK293 cells. Based on screening of ENCODE-integrated ChIP-sequencing data for candidate promoter regulating factors, these vectors were cotransfected in HeLa cells and consistently increased *ADGB* promoter-dependent luciferase activity (Fig. S6*A*). Additional cotransfection experiments with increasing amounts of GATA-3 encoding plasmids indicated GATA-3-dependent regulation of the *ADGB* promoter in a dose-dependent way (Fig. S6*B*). Next, we employed CRISPR activation (CRISPRa) technology to activate transcription at the *ADGB* promoter. CRISPRa is based on a fusion of catalytically inactive Cas9 (dCas9) with the activation domains of three potent transcription factors, VP64, p65, and Rta (dCas9-VPR), which is targeted to a specific genomic region with single guide RNA (sgRNA) to trigger locus-specific transcriptional activation (24). Several gRNAs, designed to bind upstream of the *ADGB* TSS region, were tested for their capacity to induce *ADGB* promoter-driven luciferase activity and endogenous ADGB expression. Using two gRNA sequences (termed gRNA AP-1 and gRNA AP-2), the CRISPR-based system was able to substantially induce *ADGB* promoter-driven luciferase activity in HEK293 and MCF-7 cells, validating functionality of these gRNAs (Fig. 4*B*). Similarly, the CRISPR-based system also robustly activated endogenous *ADGB* gene expression on mRNA level in both cell lines (Fig. 4*C*). Interestingly, combined transfection of gRNA AP-1 and gRNA AP-2 additively facilitated expression of the *ADGB* gene. On the protein level, a band could be observed of slightly lower molecular weight compared with predicted endogenous full-length ADGB in HEK293 (Fig. 4*D*). Immunoblotting experiments displayed similar results in MCF-7 (Fig. 4*D*). Taken together these results confirm that the upstream *ADGB* gene region possesses promoter activity.

### The *ADGB* locus contains functional enhancers

**Figure 5:**
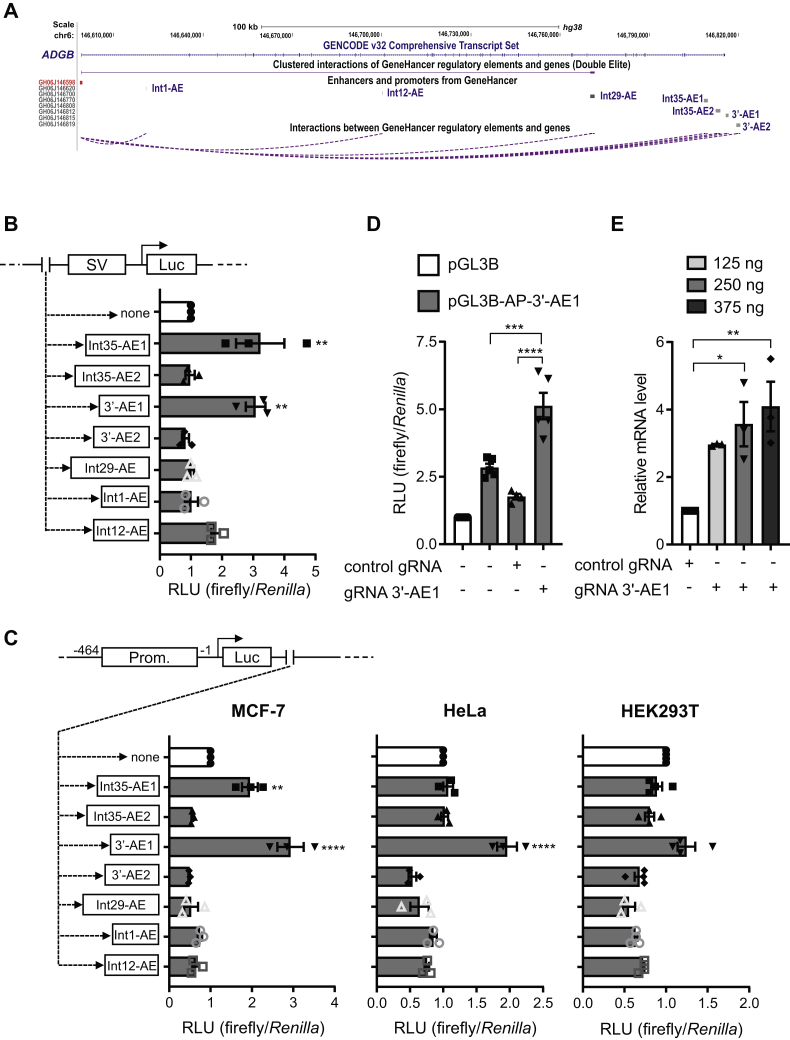
**Distal enhancers regulate *ADGB* promoter-driven gene transcription.***A*, potential *ADGB* regulatory enhancer elements interacting *via* long-range looping with the *ADGB* promoter region, derived from the GeneHancer database, are displayed in the UCSC Genome Browser. *B*, seven potential enhancer elements predicted to be in close proximity with the *ADGB* promoter region were cloned at −27 bp upstream of the SV40 promoter in a luciferase vector. These constructs were cotransfected into MCF-7 cells together with a *Renilla* control plasmid, and the effect of these enhancer elements was assessed. Results are displayed as ratios of firefly to *Renilla* luciferase activities in relative light units (RLU). The presence of Int35-AE1, 3’-AE1 and Int12-AE enhancer candidates increased SV40 promoter-driven luciferase activity (n = 3 independent experiments). *C*, the seven potential enhancer elements were cloned at 268 bp downstream of the 464 bp *ADGB* promoter-driven luciferase reporter gene. Consistent with (*C*), Int35-AE1 and 3’-AE1 sequences, but not the Int12-AE sequence, increased *ADGB* promoter-driven luciferase activity in MCF-7 cells. When tested in HeLa and HEK293T cells, 3’-AE1 sequence also displays an enhancing effect on *ADGB* promoter-driven luciferase activity (n = 3 independent experiments). *D*, dCas9-VPR-based activation of *ADGB* enhancer-dependent *ADGB* promoter (464 bp)-driven luciferase constructs using a 3’-AE1 *ADGB* enhancer-targeting gRNA (gRNA-3’-AE1) results in increased luciferase activity. *E*, HEK293T cells were transfected with dCas9-VPR along with different amounts of the 3’-AE1 *ADGB* enhancer-targeting gRNA (gRNA-3’-AE1) and relative *ADGB* transcript levels were quantified by RT-qPCR using the negative control as reference (n = 3 independent experiments). Data represent mean ± S.E.M (error bars); ∗*p* < 0.05; ∗∗*p* < 0.01; ∗∗∗*p* < 0.001; ∗∗∗∗*p* < 0.0001.

The cell-type-specific regulation of ADGB expression is likely to be under the control of multiple *cis*-regulator elements apart from the promoter alone. To further delineate the *ADGB* regulatory landscape, we mined ENCODE and ReMap-based data (25, 26) within the large *ADGB* locus. Multiple regions with strong transcription factor occupancy and DNase hypersensitivity are detectable within the *ADGB* locus (Fig. S7). Furthermore, GeneHancer-derived data suggest that the *ADGB* promoter is regulated by distal enhancer elements that come in close proximity with the promoter by long-range chromatin looping. More precisely seven different potential enhancers (GH06J146620, GH06J146700, GH06J146770, GH06J146808, GH06J146812, GH06J146815, GH06J146819) display looping to the *ADGB* promoter, based on correlations between epigenetic marks and the gene-enhancer distance algorithm implemented by the GeneHancer database (27). Five of these potential *ADGB* enhancer elements are situated within different introns of the *ADGB* gene and two are located immediately downstream of the last *ADGB* exon (exon 36) (Fig. 5*A*). All of them coincide with strong DNase hypersensitivity and substantial transcription factor occupancy, as well as frequent or occasional (depending on the enhancer) enrichment of enhancer histone marks (H3K4me1, H3K4me2, H3K27ac, and H3K9ac) in multiple mammalian cell lines (Fig. S8). Moreover, chromatin segmentation state tools suggest activity of all enhancers in several cell lines (Fig. S8). In order to experimentally investigate their functionality, we first analyzed their ability to drive SV40 promoter-dependent luciferase activity and cloned all seven potential enhancer elements (for convenience renamed as *ADGB* enhancers (AE) based on intronic or 3’ position in: Int1-AE, Int12-AE, Int29-AE, Int35-AE1, Int35-AE2, 3’-AE1, and 3’-AE2) in a pGL3Prom system (Fig. 5*B*). Reporter gene assays in MCF-7 cells displayed enhancing effects on the SV40 promoter in the presence of Int35-AE1, 3’-AE1, and Int12-AE (Fig. 5*B*), indicating that these DNA segments possess promoter-enhancing capability. Subsequently, all potential enhancer elements were cloned in the presence of the endogenous *ADGB* promoter (−1 to −464 bp upstream of the *ADGB* TSS). Corresponding with the SV40 promoter-driven luciferase assays, Int35-AE1 and 3’-AE1, but not Int12-AE, increased *ADGB* promoter-driven luciferase activity (Fig. 5*C*). 3’-AE1 displayed a more profound enhancing effect than Int35-AE1, and the effect of Int35-AE1 diminished to basal levels when the experiment was carried out in HeLa and HEK293 cells (Fig. 5*C*). Intriguingly, Int35-AE1 and 3’-AE1 display substantial sequence similarity (∼63% identity), with the entire 3’-AE1 sequence found within Int35-AE1, with some differences indicative of insertional or substitutional mutations (data not shown). Finally, we employed CRISPRa technology to activate transcription at the 3’-AE1 enhancer and validated enhancer capacities of 3’-AE1 in an endogenous context. Whereas gRNA-3’-AE1 could modestly induce 3’-AE1-dependent *ADGB* promoter-driven luciferase activity (Fig. 5*D*), gRNA-3’-AE1 also robustly enhanced endogenous ADGB mRNA levels (Fig. 5*E*), albeit to a considerably lower extent as compared with those targeting the *ADGB* promoter. Importantly, dose-dependent overexpression of the 3’-AE1 enhancer targeting gRNA increased endogenous ADGB mRNA levels accordingly. Collectively, these data indicate a complex transcriptional regulation of the *ADGB* locus.

### FOXJ1 activates the *ADGB* promoter *via* direct binding

**Figure 6:**
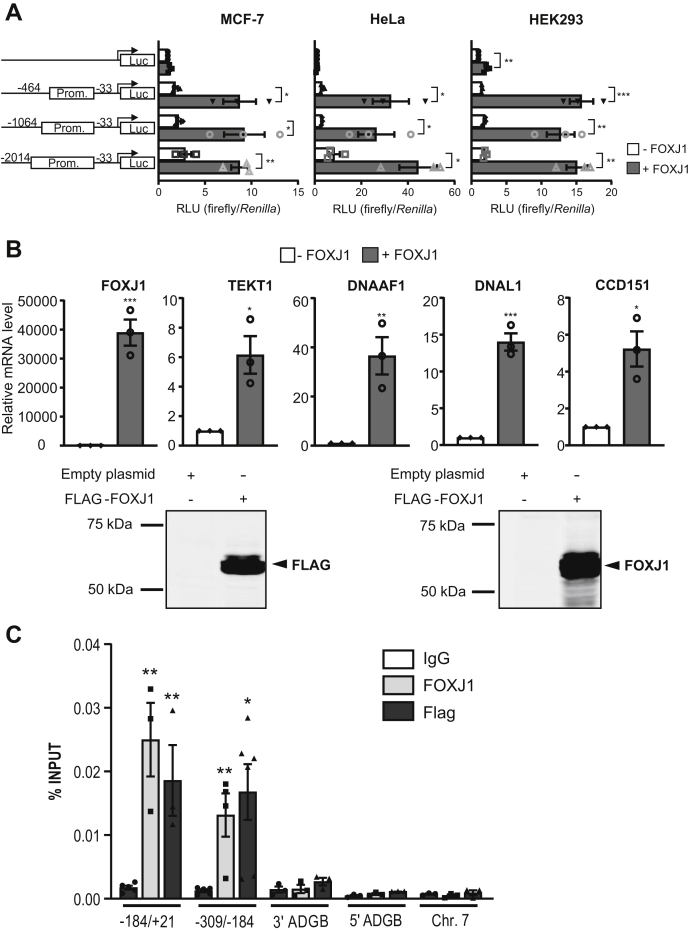
**FOXJ1 activates the *ADGB* promoter *via* direct binding.***A*, reporter gene assays of *ADGB* promoter elements in MCF-7, HeLa, and HEK293T cells with or without co-overexpression of FOXJ1 display a FOXJ1-induced activation of *ADGB* promoter-driven luciferase activity. This increase in promoter activity is seen in all promoter elements of different lengths (−33 bp to −464, −1064, and −2014 bp upstream of the *ADGB* TSS). Results are displayed as ratios of firefly to *Renilla* luciferase activities in relative light units (RLU) and normalized to the pGL3-basic vector (n = 3 independent experiments). *B*, mRNA and protein experiments of HEK293 cells transiently transfected with a FLAG-FOXJ1 vector. FOXJ1 target gene mRNA levels were measured by RT-qPCR and normalized to β-actin mRNA levels. Immunoblotting using anti-FLAG and anti-FOXJ1 antibodies also confirmed FOXJ1 overexpression. *C*, the amount of coprecipitated chromatin derived from the proximal *ADGB* promoter region using two primer pairs (covering +21 to −184 and −184 to −309 upstream of the *ADGB* TSS, selected based on the reporter gene assays), its upstream (5’) and downstream (3’) regions as well as an independent region on chromosome 7 in the *EPO* locus (64), was determined by qPCR. Data represent mean ± S.E.M (error bars); ∗*p* < 0.05; ∗∗*p* < 0.01; ∗∗∗*p* < 0.001; ∗∗∗∗*p* < 0.0001.

The ADGB expression data described above suggested a regulation of the gene by FOXJ1, an essential transcriptional regulator of motile cilia formation. To investigate the potential activation of the *ADGB* promoter by FOXJ1, we employed reporter gene assays on cloned *ADGB* promoter of varying lengths. Overexpression of FOXJ1 significantly increased *ADGB* promoter-driven luciferase activity in MCF-7, HeLa, and HEK293 cells, substantiating that FOXJ1 represents an *ADGB* promoter-targeting transcription factor (Fig. 6*A*). As FOXJ1-mediated activation of the *ADGB* promoter was observed in promoter segments of different but overlapping lengths, the binding site of FOXJ1 might be situated in the smallest −33 to −464 bp region, present in all three of the cloned *ADGB* promoter constructs, while the presence of multiple interaction sites along the longest −33 to −2014 bp fragment cannot be excluded. To analyze FOXJ1-DNA binding to the endogenous *ADGB* promoter region, ChIP assays were performed using anti-FLAG and anti-FOXJ1 antibodies in HEK293 cells transiently transfected with FLAG-tagged FOXJ1 constructs. To control for FOXJ1 overexpression, we analyzed endogenous mRNA levels of four established FOXJ1 target genes (Fig. 6*B*). Whereas FOXJ1 expression levels were strongly upregulated, also mRNA levels of its target genes DNAAF1, TEKT1, CCDC151, and DNAL1 were robustly induced following transient transfection of FOXJ1. Similarly, immunoblotting confirmed expression of the chimeric protein using anti-FLAG and anti-FOXJ1 antibodies (Fig. 6*B*). Quantitative ChIP analysis revealed more than tenfold FOXJ1 enrichment at the endogenous promoter region compared with the IgG control using two primer pairs spanning the upstream proximal *ADGB* region (Fig. 6*C*), but not at two more distal upstream and downstream regions, neither at an independent region on chromosome 7 (Fig. 6*C*). Consistently, no binding was observed in nontransfected cells (Fig. S9). These results confirm that FOXJ1 also binds to the endogenous *ADGB* promoter.

### Evolutionary conserved nucleotides within −71 ± 30 bp upstream of the *ADGB* gene are required for FOXJ1 binding.

**Figure 7:**
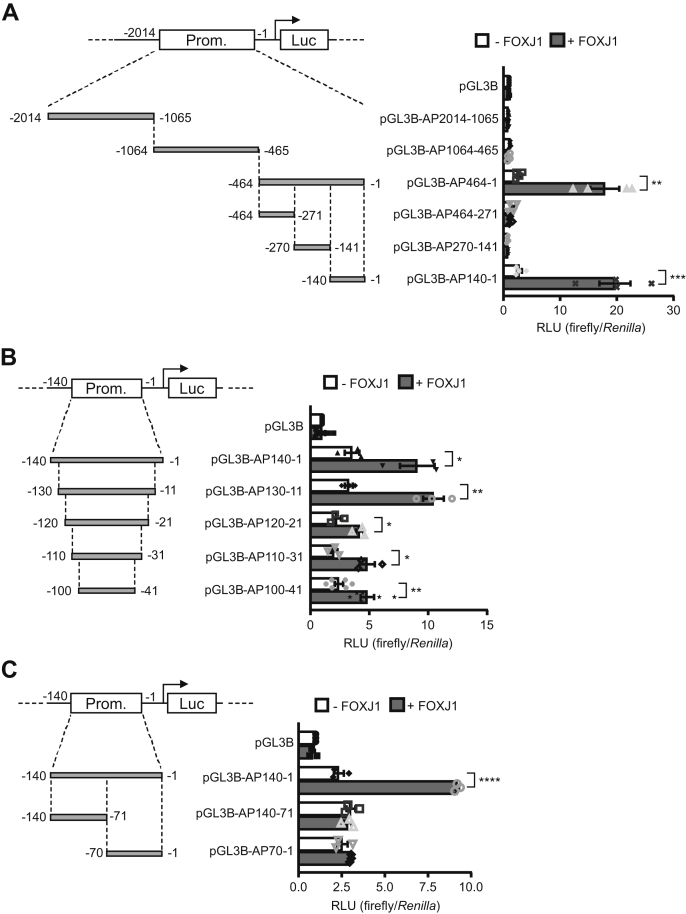
**The proximal *ADGB* promoter confers FOXJ1-mediated increase in luciferase activity.***A*, the longest *ADGB* promoter (AP) element, AP2014 (−33 to −2014 bp upstream of TSS), displaying FOXJ1-induced promoter activity was first divided into three nonoverlapping segments (−1065 to −2014 bp, −465 to −1064 bp, and −1 to −464 bp upstream of the *ADGB* TSS) and cloned individually upstream of the firefly luciferase gene. Only the segment −1 to −464 bp upstream of the *ADGB* TSS displays FOXJ1-induced promoter activity, and no induction is observed with inserts covering the −465 to −2014 bp upstream of the *ADGB* TSS (n = 4 independent experiments). Schematic representation of different *ADGB* promoter segments is shown with numbers representing positions corresponding to the first nucleotide of the TSS. The −1 to −464 bp *ADGB* promoter construct was further subdivided into three nonoverlapping segments (−271 to −464 bp, −141 to −270 bp, and −1 to −140 bp upstream of the *ADGB* TSS) and cloned individually upstream of the firefly luciferase gene. From those only insert −1 to −140 bp displays FOXJ1-induced promoter activity (n = 4 independent experiments). *B*, the −1 to −140 bp *ADGB* promoter construct was sequentially reduced at both the 5’- and 3’- ends by 10 bp, 20 bp, 30 bp, and 40 bp, to generate smaller segments of −11 to −130 bp/−21 to −120 bp/−31 to −110 bp/−41 to −100 bp upstream of the *ADGB* TSS. All segments display FOXJ1-induced promoter activity including the smallest 60 bp segment of −41 to −100 bp (n = 3 independent experiments). *C*, −1 to −140 bp *ADGB* promoter element was subdivided into two segments of 70 bp, −1 to −70 bp and −71 to −140 bp. In both constructs, FOXJ1-induced promoter activity was abolished (n = 3 independent experiments) suggesting a disruption to the FOXJ1 binding site or missing binding sites for proper FOXJ1 interaction.

**Figure 8:**
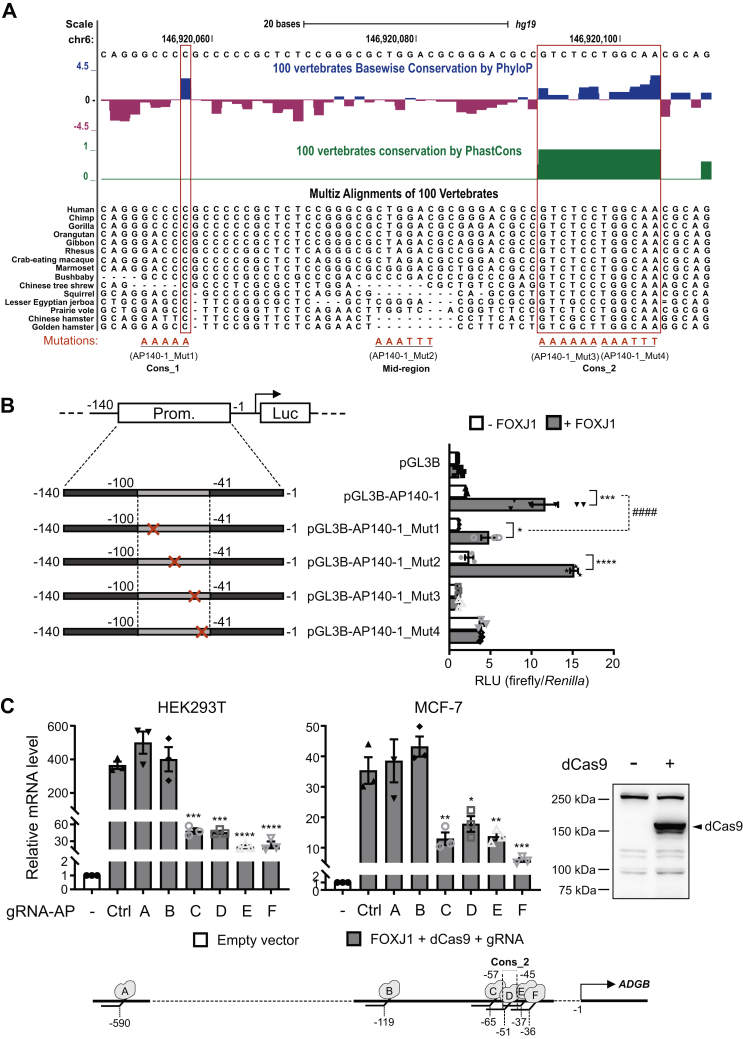
**Conserved nucleotides within −71 ± 30 bp upstream of *ADGB* TSS are crucial for FOXJ1 binding.***A*, UCSC Genome Browser output (*hg19*) of evolutionary conserved nucleotides within −41 bp to −100 bp upstream of the *ADGB* TSS based on a subset of vertebrate sequences extracted from the 100-MULTIZ whole-genome multiple sequence alignment algorithm. Basewise conservation scored by PhyloP indicates conserved and variable nucleotides in blue and red bars, respectively. Highly conserved nucleotides, also supported by the PhastCons track within this region of the *ADGB* promoter are boxed in red. Mutation strategy of conserved nucleotides is illustrated at the bottom. *B*, substitution-based mutation at −96 to −92 bp (Mut1), −57 to −52 bp (Mut3), and −51 to −46 bp (Mut4) results in loss of FOXJ1-dependent increase in *ADGB* promoter activity. Whereas the mutation at −73 to −68 bp (Mut2) did not abolish the FOXJ1-mediated activation (n = 3 independent experiments). *C*, sgRNA-mediated docking of dCas9 onto the *Cons2* region in the *ADGB* promoter results in reduced FOXJ1-dependent activation of ADGB expression while more remote control sgRNAs display no effect in HEK293 and MCF-7 cells (n = 3 independent experiments). gRNA positions are schematically represented. Immunoblotting analysis using a Cas9 antibody controlled for dCas9 overexpression. Data represent mean ± S.E.M (error bars); ∗*p* < 0.05; ∗∗*p* < 0.01; ∗∗∗*p* < 0.001; ∗∗∗∗*p* < 0.0001.

To narrow down the search for the FOXJ1-binding site within the *ADGB* promoter, we further dissected the longest *ADGB* promoter segment (−33 to −2014 bp) into three nonoverlapping segments (Fig. 7*A*) indicating the absence of FOXJ1-mediated activation in more distal *ADGB* promoter segments. In contrast, FOXJ1 overexpression significantly increased the promoter activity of segment −1 to −464 bp, suggesting that the FOXJ1-binding site is limited to this segment closest to the *ADGB* TSS (Fig. 7*A*). Further refinement of the FOXJ1 responsive region by dividing the −1 to −464 bp segment into three nonoverlapping segments indicated that only the −1 to 140 bp segment closest to the *ADGB* TSS was highly activated by FOXJ1 (Fig. 7*A*). Next, we further trimmed down the length of the −1 to −140 bp segment from both the 5’- and 3’- ends by 10 bp, 20 bp, 30 bp, and 40 bp (Fig. 7*B*). The incremental reduction of −1 to −140 bp segment from both ends at 10 bp intervals did not abolish the FOXJ1-mediated increase in *ADGB* promoter-driven luciferase activity, although a drop in the luciferase signals could be observed in the smaller *ADGB* promoter fragments of −21 to −120 bp/−31 to −110 bp/−41 to −100 bp (Fig. 7*B*). These results suggest that the FOXJ1 interaction site remains in all of these segments. When the −1 to −140 bp fragment was divided into two equally long, nonoverlapping parts, FOXJ1-mediated increase in promoter activity was abolished (Fig. 7*C*). This indicated that the mid-region of −70 bp might be important for FOXJ1 interaction, or the −1 to −70 bp and −71 to −140 bp divided segments each contain part of the FOXJ1 interaction site. Multiple sequence alignments of several vertebrate species based on the MULTIZ algorithm within −71 ± 30 bp upstream of the *ADGB* TSS indicated the presence of evolutionary conserved nucleotides within this segment of the promoter (Fig. 8*A*). To further fine-map the FOXJ1 binding site, we separately mutated three regions within the −71 ± 30 bp *ADGB* promoter region, one containing a single conserved nucleotide (termed *Cons1*), one region displaying evidence of evolutionary constraint as reflected by phyloP and phastCons scores (termed *Cons2*), and one within the mid-point at −71 bp, as the separation of this region resulted in abolished FOXJ1-mediated activation (Fig. 7*C*). It was suggested that Fox TF-binding sites are approximately 8 to 10 bp in length (28). Therefore, we mutated these regions by substitution of 5 to 6 residues with tandem A and/or T, which are likely to be sufficient to disrupt potential FOXJ1 binding (Fig. 8*A*). For *Cons2*, two separate mutants of proximal and distal parts were constructed due to the 12 bp size of the conserved region. Interestingly, mutations on *Cons1* and *Cons2* abolished the FOXJ1-mediated increase in *ADGB* promoter-driven luciferase activity, whereas the mid-point mutation did not. (Fig. 8*B*). This result suggests that both conserved regions (*Cons1* and *Cons2*) within the promoter might be important for FOXJ1 interaction. Moreover, these conserved regions are probably mutually dependent on each other to mediate FOXJ1 interaction as the absence of either part disrupted FOXJ1-mediated activation on the *ADGB* promoter. This might also explain the abolished FOXJ1 activation in pGL3B-AP70-1 and pGL3B-AP140-71 (Fig. 7*C*), as both of these interdependent FOXJ1 interaction sites were separated in these constructs. Similar findings were obtained with an *ADGB* promoter fragment of reduced size (Fig. S10). Finally, to independently validate the FOXJ1-binding site endogenously, we employed the CRISPR/dCas9 approach with *ADGB* promoter gRNAs to block the genomic-binding site in the presence of exogenous FOXJ1. The docking of a dCas9 variant uncoupled from VPR onto the putative FOXJ1-binding site hinders the interaction of FOXJ1 with the *ADGB* promoter. Similar to the negative control gRNA cotransfection of two more remotely located gRNAs (−590 and −119 bp upstream of the *ADGB* TSS) had no effect on the FOXJ1-dependent activation of ADGB expression. In contrast, transfection of three different *Cons2*-overlapping gRNAs as well as a gRNA 8 bp upstream of *Cons2* all significantly reduced FOXJ1-mediated increase of ADGB transcription in both HEK293 and MCF-7 cells (Fig. 8*C*). Taken together, these data strongly indicate that the FOXJ1 interaction site is located within −71 ± 30 bp upstream of the *ADGB* TSS and involves two evolutionary conserved regions.

### Overexpression of FOXJ1 and RFX2 induces endogenous ADGB mRNA levels

**Figure 9:**
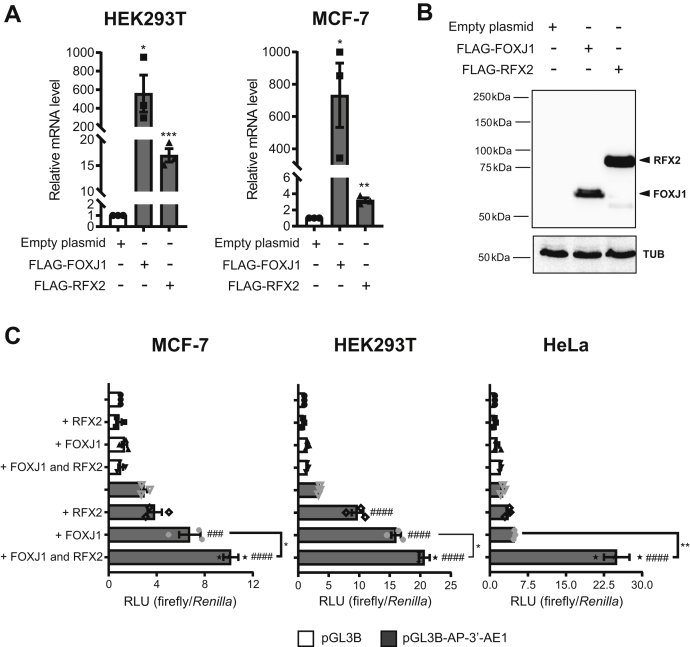
**FOXJ1 and RFX2 transcription factors induce endogenous ADGB transcription and cooperate to regulate ADGB expression in the presence of the 3’-AE1 enhancer element.***A*, HEK293T and MCF-7 cells were transiently transfected with a FLAG-tagged FOXJ1 or RFX2 expressing plasmid and ADGB mRNA levels were measured with RT-qPCR. ADGB expression levels were normalized to β-actin and displayed as relative values to cDNA of HEK293T or MCF-7 cells transfected with equal amount of empty vector. *B*, FLAG-tagged FOXJ1 and RFX2 plasmid-derived expression levels were controlled by immunoblotting using an anti-FLAG antibody. Tubulin (TUB) was used as loading control. *C*, 3’-AE1 *ADGB* enhancer-dependent *ADGB* promoter-driven reporter gene assays. Following overexpression of FOXJ1, FOXJ1-mediated activation of the *ADGB* promoter activity is observed as expected from previous experiments. Co-overexpression of FOXJ1 and RFX2 shows synergistic activation of the *ADGB* promoter construct in the presence of 3’-AE1 enhancer element (n = 3 independent experiments). Similar experiments were performed in HEK293T and HeLa (n = 3 independent experiments). Data represent mean ± S.E.M (error bars); ∗*p* < 0.05; ∗∗*p* < 0.01. ###*p* < 0.001; ####*p* < 0.0001.

To investigate the effect of FOXJ1 in the regulation of ADGB expression, we overexpressed this transcription factor in HEK293 cells, expressing no endogenous ADGB as well as very little endogenous FOXJ1, and subsequently measured endogenous ADGB mRNA expression levels. Overexpression of FOXJ1 in HEK293 cells profoundly increased expression levels of endogenous ADGB (Fig. 9*A*), further confirming a FOXJ1-dependent regulation of ADGB expression. Given the cooperative functional association between FOXJ1 and RFX2, another essential transcriptional regulator of ciliogenesis (28), we also assessed a potential RFX2-dependent regulation of ADGB. Consistent with the RNA-Seq results of *Rfx2*-deficient mice suggesting a RFX2-dependent regulation of ADGB transcription (29), also overexpression of RFX2 in HEK293 cells increased endogenous ADGB expression levels (Fig. 9*A*), albeit to a lower extent than FOXJ1. FLAG-tag-based immunoblotting experiments excluded that this discrepancy arose from differences in plasmid expression (Fig. 9*B*). As the 36-exon containing ADGB pre-mRNA might be alternatively spliced to produce different variants of the protein, we confirmed these results by employing multiple exon–exon primer pairs for RT-qPCR across the whole ADGB transcript (Fig. S11). Similar results were obtained in MCF-7 cells (Fig. 9*A*). Collectively, these findings indicate that ADGB is a downstream effector of the two master regulators of ciliogenesis FOXJ1 and RFX2, further suggesting a potential role of ADGB in the formation and/or function of cilia.

### FOXJ1 and RFX2 synergistically activate the *ADGB* promoter in the presence of the 3’-AE1 enhancer in reporter assays

In order to understand the role of RFX2 in regulating the *cis*-regulatory elements of the *ADGB* gene, we employed reporter gene assays to elucidate the interaction of RFX2 on these regulatory elements. Subsequently, we postulated that RFX2 might be binding to the enhancer elements that are in close proximity with the promoter. In order to exclude the possible influence of the endogenous *ADGB* promoter on the readout, we co-overexpressed RFX2 with heterologous SV40 promoter-driven luciferase reporter constructs coupled with *ADGB* enhancers. Our results displayed no RFX2-mediated activation of promoter activity despite the presence of enhancer elements (Fig. S12), suggesting that RFX2 might not interact with any of these enhancer elements in a direct manner. Next, we examined the role of RFX2-mediated activation of *ADGB* regulatory elements in the presence of FOXJ1, *ADGB* promoter and enhancer elements. Reporter assays illustrated no difference in *ADGB* promoter activity across all coupled enhancers with RFX2 overexpression alone (Fig. S12). However, under FOXJ1 overexpression conditions, FOXJ1-mediated activation in each of the *ADGB* promoter-driven reporter constructs could be observed. Interestingly, co-overexpression of FOXJ1 and RFX2 displayed additive activation of *ADGB* promoter activity only in the presence of 3’-AE1 enhancer element (Fig. 9*C*), but none of the other *ADGB* enhancers (Fig. S12), indicating that the presence of FOXJ1 and the 3’-AE1 enhancer is a prerequisite for RFX2-mediated activation of the *ADGB* promoter. Similar experiments in two independent cell lines, HEK293 and HeLa, validated the 3’-AE1 enhancer-dependent regulation. Whereas this regulation was additive in HEK293, a synergistic regulation could be observed for HeLa cells (Fig. 9*C*). As this FOXJ1-RFX2 synergy was not observed in the sole *ADGB* promoter-driven reporter constructs, the 3’-AE1 enhancer is crucial in mediating the synergistic effect. Hence, these experiments strongly suggest that RFX2 supports the FOXJ1-mediated regulation of ADGB expression with the presence of the essential 3’-AE1 enhancer.

### Ectopic ADGB overexpression promotes ciliogenesis

**Figure 10:**
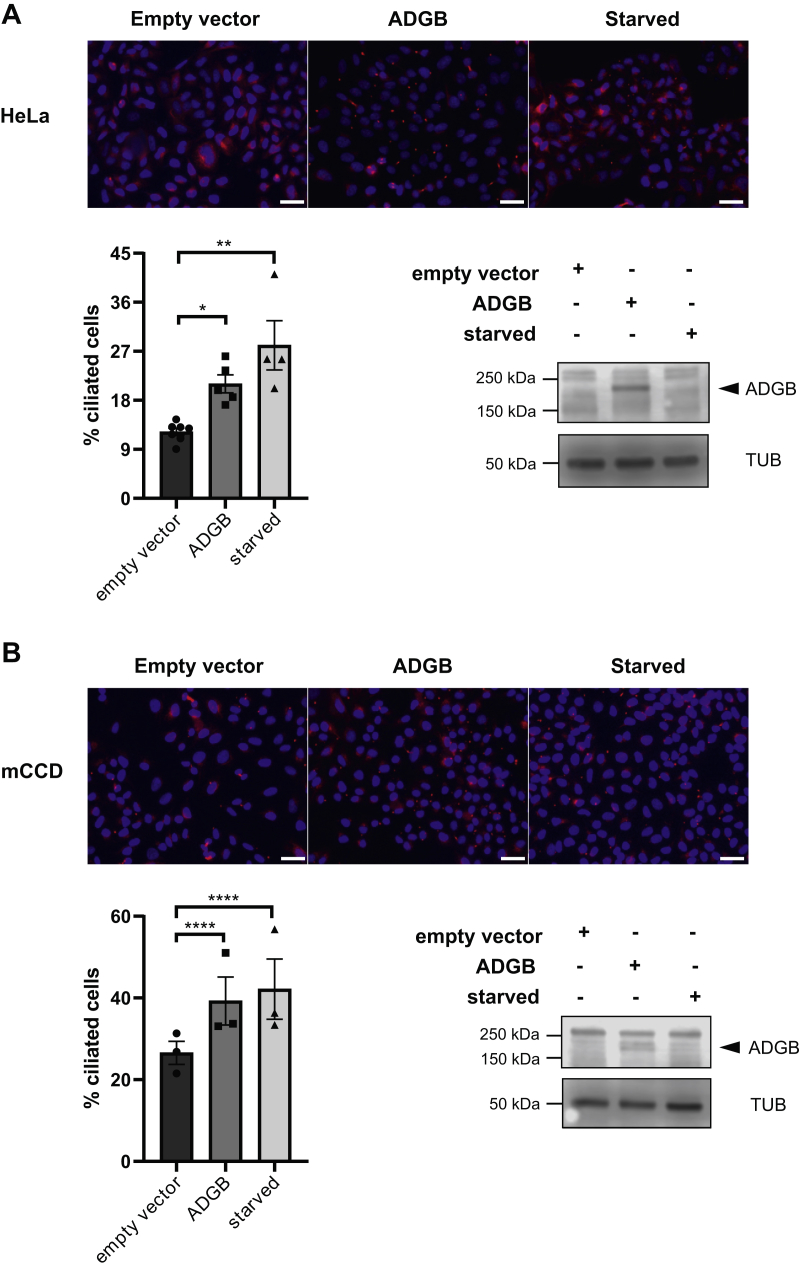
**ADGB overexpression promotes ciliogenesis in two independent cell lines.***A*, representative pictures of acetylated α-tubulin (*red fluorescence*) and DAPI (*blue fluorescence*) in HeLa cells under basal conditions transfected with empty vector, following transfection with 2 μg ADGB or following 24 h serum starvation (starved), and corresponding cilia quantifications (expressed as percentage of ciliated cells) (n = 3–4 independent experiments, 3–5 pictures were counted for each condition and per experiment). Scale bar represents 100 μm. Overexpression of ADGB was verified by immunoblotting. Tubulin (TUB) was used as loading control. *B*, Similar representative immunofluorescence pictures of acetylated α-tubulin, corresponding cilia quantifications and immunoblotting following transfection with 2 μg ADGB in mCCD_cl1_ cells. Scale bar represents 100 μm. Data represent mean ± S.E.M (error bars); ∗*p* < 0.05; ∗∗∗*p* < 0.001; ∗∗∗∗*p* < 0.0001.

As FOXJ1 and RFX2 represent both critical regulators of ciliogenesis, a potential role of ADGB in the formation and/or function of cilia is plausible. To explore a putative role for ADGB in ciliogenesis, we examined ADGB requirements in cilia formation in cellular models. Due to the lack of cellular models with robust endogenous ADGB expression levels, we performed ADGB overexpression. Immunostaining with antiacetylated tubulin revealed that the number of cilia was substantially increased following ADGB overexpression in human HeLa cells (Fig. 10*A*). Cilia formation was similarly increased in ciliated mouse cortical collecting duct cells following overexpression of ADGB, almost comparable with serum-starved induction of ciliogenesis (Fig. 10*B*). These findings are in perfect agreement with the expression analyses presented above and collectively suggest that ADGB is associated with ciliogenesis and could play an evolutionarily conserved role in the formation and/or maintenance of cilia.

## Discussion

ADGB, the fifth member of the mammalian globin family (5), is a chimeric protein with an unusual, embedded globin domain that is circularly permutated and exhibits hallmarks of a hexacoordinated heme-binding scheme (30). Intriguingly, abundant expression of ADGB in various species seemed to be restricted to the testis tissue (hence its name) and, more specifically, to postmeiotic stages of spermatogenesis. The function of ADGB, however, has remained unclear. Since the gene’s initial description (5), a wealth of transcriptome data has been produced by the scientific community, facilitating a re-evaluation of ADGB’s expression profile. Our extended expression analysis of ADGB in mammalian tissues based on bulk and single-cell RNA-Seq data, including confirmation by RT-qPCR and immunohistochemistry, revealed that ADGB is consistently detected in cells carrying motile cilia or flagella. Specifically, substantial ADGB expression was also observed in the *female* reproductive tract, which obviously adds a completely new perspective on its original designation suggesting a predominant role in males. Prominent ADGB mRNA expression was also observed in the lung epithelial cells and in the brain ependymocytes and α-tanycytes, altogether possessing motile cilia. A particularly interesting result was the bioinformatically inferred correlation of ADGB expression with the master transcription factor of ciliogenesis, Foxj1 (see below). Additional independent support for a ciliogenesis-associated role of ADGB came from the CiliaCarta database, a multiomics-based comprehensive ciliary compendium suggesting that ADGB is a human ciliome component with a high rank and probability score (31). A recent evolutionary proteomics approach (32) indicated that the association of ADGB with ciliary structures may be phylogenetically ancient and can even be detected down to the flagellum-containing choanoflagellates, which is in agreement with our own phylogenetic reconstruction of ADGB ancestry (5). In fact, such a phylogenetic perspective lends additional weight to the proposed functional association of ADGB and cilia: despite the presence of numerous globin genes in their genomes (33, 34, 35), *Drosophila melanogaster* and *Caenorhabditis elegans* both are missing orthologues of ADGB (5), and both organisms are devoid of motile cilia on their somatic cells (36). Movement of spermatozoa in *C. elegans* is achieved *via* pseudopods, which carry no resemblance to classical flagella (37). *Drosophila*, on the other hand, still develops motile flagella during spermatogenesis (38). However, the fly harbors two testis-specific globins, which are not phylogenetically related to ADGB (39). In expression analyses, these *Drosophila* testis globins were correlated with genes characterized by GO-terms such as sperm axoneme assembly and motility (40). It is therefore tempting to speculate that these globins at least partially compensate for the loss of ADGB in the fruit fly. In addition, ADGB orthologues appear to be missing in the phylum of crustaceans (5), which form specialized, mostly immobile spermatozoa (41).

The observed strictly cell-specific expression pattern of ADGB prompted us to comprehensively investigate its transcriptional regulation. In accordance with a suggested role in ciliogenesis, our experimental data provided direct evidence that the *ADGB* gene is indeed regulated by FOXJ1. For further confirmation, we inspected transcriptome screens of FOXJ1 knockout and overexpression models in mouse, zebrafish, and frogs (42, 43, 44, 45) and detected consistent evidence for FOXJ1-dependent expression levels of *ADGB* in those data sets. Furthermore, a recent *in silico* study of FOXJ1-mediated regulatory and signaling networks predicted *ADGB* as one of the direct FOXJ1-regulated genes (46). During spermatogenesis, FOXJ1 expression coincides with the timely stages of flagella formation where it probably orchestrates the expression of genes essential for flagella biogenesis (47, 48). The broader role of FOXJ1 as the master regulator of motile ciliogenesis has been reported as well (10). In addition to FOXJ1-dependent expression, our reporter gene assays and overexpression experiments revealed that the *ADGB* gene is also robustly regulated by RFX2, a transcriptional activator of spermiogenesis (29). Again, this is in strong agreement with transcriptome data from *Rfx2*-deficient mice (29) showing Rfx2-dependent expression of *Adgb*. The latter study also listed Rfx2 binding to the mouse *Adgb* locus in ChIP-sequencing experiments.

A recent study reported that the cooperation of FOXJ1 and RFX2 has a prominent role at promoters of ciliary genes compared with other established cilia transcription factors. Both transcription factors were found to be positioned at the anchor end point of chromatin loops, where RFX2 was suggested to act as a scaffolding factor to stabilize the distal enhancer element with the proximal promoter, thus bringing the enhancer-binding FOXJ1 closer to the promoter (28). Consistent with these findings, our study describes that the remote enhancer 3'-AE1, located downstream of the *ADGB* gene, is important for RFX2 to cooperate in synergy with FOXJ1 in activating *ADGB* promoter-driven luciferase activity. It is thus likely that RFX2 acts as the mediator that enables the connection between enhancer 3'-AE1 and the *ADGB* promoter, which could explain the lack of RFX2-mediated transactivation of 3'-AE1-dependent SV40-driven luciferase activity. Correspondingly, RFX2 also shows little to no activation on enhancer 3'-AE1 coupled with the *ADGB* promoter, suggesting a possible scaffolding promoter–enhancer mediator role with no transcriptional activity. It remains to be determined if, in an endogenous genomic context, RFX2 is crucial to establish the connection between the *ADGB* promoter and enhancer 3'-AE1, explaining the modest upregulation of endogenous ADGB upon RFX2 overexpression. In slight contrast to the study of Quigley and Kintner (28), our findings from reporter assays indicate a functional interaction of FOXJ1 at the *ADGB* promoter rather than at its distal enhancers. In our reporter gene studies, RFX2 synergistically activates the *ADGB* promoter with FOXJ1 only in the presence of the distal enhancer 3'-AE1, which is in line with Quigley and Kintner (28) and indicates that FOXJ1 is stabilized at promoters of cilia genes through cooperative interactions with RFX2.

Our study provided extensive efforts in refining the interaction site(s) of FOXJ1 on the *ADGB* promoter, which enabled the identification of evolutionarily conserved nucleotides that are crucial for FOXJ1-mediated activation of the *ADGB* promoter. The FOXJ1-binding motif has not been fully annotated so far. A single computational study deduced the preferential binding of FOXJ1 to the consensus sequence NNN[G/A]TAAACAAANNN, with N representing any nucleotide (46). However, only a sole motif with this consensus can be found within the −2014 to −1065 bp upstream *ADGB* promoter sequence, whereas motifs with less stringent sequence similarity can be found within −465 to −2014 bp upstream of the *ADGB* TSS. From our experimental data, this part of the *ADGB* promoter shows no FOXJ1-mediated activation. In another study employing *Xenopus laevis*, an RFX-based analysis for binding motifs in the promoters of multi-cilia-related genes has proposed a consensus binding motif in human orthologs (TTCCTGGAAAC). Although this motif was suggested to be the binding site for RFX TFs, also enrichment of FOXJ1 in this RFX-motif was reported, probably due to cobinding of FOXJ1 and RFX factors (28). Strikingly, this binding motif displays very strong sequence similarity to the *Cons2* region in the *ADGB* promoter whose mutation abolished FOXJ1-mediated activation. Therefore, our conservation-based analysis of FOXJ1 binding on the *ADGB* promoter is independently validated by the *in silico* analysis of FOXJ1-enriched motifs.

The transcription factor p73 plays a major role in ciliogenesis and acts upstream of FOXJ1 and RFX2 (49). Nemajerova *et al.* (50) reported that TP73 deficiency broadly attenuates ciliary gene expression by transcriptome analysis of mouse tracheal epithelial cells (mTEC) derived from WT and TAp73-deficient mice. In line with a role of ADGB in ciliogenesis, mTEC air–liquid interfaces (ALI) cultured for 0, 4, 7, and 14 days of differentiation displayed increasingly abundant ADGB expression. Moreover, RNA-Seq-based transcriptome analysis of ALI cultured mTECs derived from TAp73-deficient mice showed significantly reduced ADGB levels, further substantiating a Tp73-dependent regulation, either directly or more likely *via* its downstream targets Rfx2/Foxj1, which both displayed downregulated mRNA levels in TAp73 knockout mice. Simultaneously, ChIP-Seq experiments (50) linked p73 directly to FOXJ1/RFX2 and, most interestingly, revealed p73 binding to the distal *ADGB* enhancer 3'-AE1 of the *ADGB* locus. This exquisite dependency of *ADGB* expression on ciliogenesis-associated transcription factors is accompanied by open chromatin marks at the *ADGB* promoter. Surprisingly, this epigenetic feature was also observed in a variety of transcriptionally silent cell types, which—for unknown reasons—may thus contain poised promoters.

In conclusion, our study provides first-time evidence that ADGB is specifically expressed in cell types with motile cilia, that its cellular role is most probably associated with cilia biogenesis and function, and that it is a direct regulatory target of FOXJ1 in a complex regulatory landscape. The exact role of ADGB in ciliogenesis remains to be established. Future investigations involving the generation of new animal models with conditional knockout of *Adgb* in ciliated tissues will hopefully reveal the intriguing physiological role of ADGB in cilia formation and the contribution of FOXJ1- and RFX2-dependent gene regulation.

## Experimental procedures

### Analysis of bulk RNA sequencing, single-cell RNA sequencing, and microarray data

Publicly available transcriptome raw data (Table S1) were downloaded from either NCBI or ENA web servers (https://www.ncbi.nlm.nih.gov/sra; https://www.ebi.ac.uk/ena). We only included data from Illumina machines with a minimal read length of 50 nt. For organism-wide gene expression (PRJEB6971 (Hsa; https://www.proteinatlas.org/) and PRJNA263600 (Bta)), we focused on data from large sequencing consortia, to ensure comparability. Trimming parameters were assessed for each data set *via* inspection with FastQC (https://www.bioinformatics.babraham.ac.uk/projects/fastqc/). Adapter and quality trimming were performed with BBDuk (https://sourceforge.net/projects/bbmap/). We did not apply the same trimming parameters to all the data sets to account for differences in quality and sequencing length; however, differential expression analysis was only performed on data sets from the same study with the same trimming mode. After processing, the reads were mapped against the corresponding reference genomes of either *Homo sapiens* (GRCh 38) or *Bos taurus* (Bta UMD3.1) with HISAT2 (51). Calculation of *transcripts-per-million* values (TPM) was performed with StringTie (52) in guided mode. Differential gene expression analysis (Bta) as well as hierarchical clustering of genes (Hsa) was done in R (https://www.r-project.org/) with the help of Bioconductor’s DESeq2 package (53). Gene ontology analysis was performed with WebGestalt 2019 (54). Mapped single-cell data (Table S1) were downloaded from NCBI as UMI count tables. In the lung data set, we only included mice #2 and #3, which share the same genetic background, retaining 5010 out of 7193 cells from the original analysis (16). Graph-based clustering of cells was performed using Seurat 3 (55). Our clusters were mostly in accordance with the ones published before, although we did not prefilter for contaminating cell types such as macrophages to ensure impartial analysis.

### Animals

Cattle tissue was obtained from young females (heifers) immediately after slaughter in a regional commercial slaughterhouse. One uterine horn was opened lengthwise and the endometrial portion of the uterus was dissected. Tissue was either flash-frozen on dry ice (RNA extraction) or fixed in 4% para-formaldehyde (immunofluorescence).

### RNA extraction and reverse-transcription quantitative PCR (RT-qPCR)

RNA extraction of cattle tissues was performed from snap-frozen samples with the RNeasy Plus Universal Mini kit (Qiagen) according to the instructions of the manufacturer. Approximately 50 mg of tissues was grinded and homogenized with a MiniLys (Precellys) system using mixed ceramic beads (Precellys Lysing CKMix). Difficult tissues such as endometria were pregrinded manually on dry ice with a cool scalpel. RNA was eluted in nuclease-free water. RNA quality was assessed with a Bioanalyzer (Agilent), and only samples with RIN >7 were used for further analysis. RNA was quantified *via* Qubit measurement using the Broad Range RNA Assay Kit (Thermo Fisher) and was stored at −80 °C until further use. To confirm the bioinformatical findings, we performed reverse-transcription quantitative PCR (RT-qPCR) on tissues from the female reproductive tract of cattle. 1000 ng of total cattle RNA per sample was used for reverse transcription with the SuperScript III enzyme (10,000 units per assay; Invitrogen) using an Oligo-dT primer. In the absence of validated reference genes, the amount of mRNA expression was normalized on the adjusted total amount of carefully quantified total RNA. To additionally control for differences in cDNA synthesis, 100 ng of *Drosophila* total RNA was added to the reaction as a spike-in control. RT-qPCR was carried out using GoTaq qPCR Master Mix (Promega) on the ABI Prism 7500 Fast Detection System (SDS, Applied Biosystems) and interpreted using 7500 Software Version 2.3. Quantification of ADGB-cDNA molecules was done in absolute numbers applying a calibration standard curve with known amounts of target PCR product, previously cloned into the pGEM T-easy vector system (Promega). Foxj1 and Dnah5 expressions were measured as relative values only and the sample with the highest expression was set to 100%. Copies of the *Drosophila* Globin 1 (Glob1) cDNA of the internal control were measured in parallel to identify samples with substandard reverse transcription. All primers used are listed in Table S2. For HEK293 cells, total RNA was extracted as previously described (56). Total RNA (2 μg) was reverse transcribed (RT) using the Prime Script RT reagent kit (Takara Bio USA) and cDNA levels were estimated by qPCR using the primers listed in Table S2 and a KAPA SYBR FAST qPCR reagent kit (Sigma-Aldrich) in a CFX96 C1000 Thermal Cycler (BioRad). Transcript levels were calculated as described before (57) and displayed as relative expression levels.

### Expression plasmid constructs

pENTR233-FOXJ1 entry clone obtained from the DNASU plasmid repository (58) and pcDNA3.1/nV5-DEST mammalian expression vector were Gateway-recombined according to the manufacturer’s instruction (Invitrogen) to generate a pcDNA3.1-nV5-FOXJ1 expression vector. The pcDNA3.1-HA-RFX2 plasmid was a generous gift from Prof. Zijie Sun (Stanford). N-terminally FLAG-tagged FOXJ1 and RFX2 were cloned by amplifying FOXJ1 and RFX2 genes using primer pairs with *Sal*I/*Kpn*I and *Bgl*II/*Sal*I overhangs, respectively as listed in Table S2. Amplicons were subsequently digested with their respective restriction enzymes as designed on the primers and ligated into linearized pFLAG-CMV-6a vector to generate pFLAG-FOXJ1 and pFLAG-RFX2.

### Luciferase constructs

*ADGB* promoter elements spanning from −33 bp to −464 bp, −1064 bp, and −2014 bp upstream of the *ADGB* transcriptional start site (TSS) were cloned into a pGL3-Basic (Promega) vector at −67 bp upstream of the firefly luciferase reporter gene. Promoter elements were amplified from a pool of genomic DNA extracted from three human cell lines, MCF-7, HEK293T, and Hep3B cells, by PCR using the primer pairs described in Table S2. PCR amplicons were digested with their respective restriction enzymes and ligated into linearized pGL3-Basic (Promega) vector digested with *Kpn*I and *Nhe*I. Evolutionarily conserved nucleotides within −71 ± 30 bp upstream of the *ADGB* TSS were mutated into a tandem of 5X or 6X A or T (or both) using oligonucleotide-based cloning of mutant promoter fragments into pGL3-Basic (Promega) vector at −67 bp upstream of a firefly luciferase gene. The wild-type sequences were mutated as indicated in Table S2. Prior to cloning, phosphorylated oligo duplexes with designed 5’-*Kpn*I overhang and 3’-*Nhe*l overhang were generated by incubating 0.5 μM of synthesized complementary oligo strands in T4 DNA ligase buffer (ThermoScientific) (40 mM Tris-HCl, 10 mM MgCl_2_, 10 mM DTT, 500 μM ATP) with T4 polynucleotide kinase (ThermoScientific) at 37 °C for 1 h, followed by heating to 95 °C for 5 min and slow cooling at the rate of −5 °C min^−1^ to 10 °C. Oligo duplexes were subsequently ligated into *Kpn*I and *Nhe*I digested pGL3-Basic vector backbone. Potential *ADGB* intronic and 3’ enhancer elements from the GeneHancer database (27) were cloned in a pGL3-SV40 vector (Promega), at −27 bp upstream of the SV40 promoter. These putative enhancer elements were amplified from genomic DNA, by PCR using the primer pairs as described in Table S2. PCR amplicons were digested with their respective restriction enzymes and ligated into linearized pGL3-SV40 (Promega) vector digested with *Kpn*I and *Nhe*I or into the linearized *ADGB* promoter containing pGL3B-AP464 vector, at 269 bp downstream of the firefly luciferase gene, digested with *BamH*I.

### Mammalian cell culture and DNA transfection

MCF-7, HEK293T, and HeLa cells were cultured and maintained in DMEM (Thermo Fisher) medium supplemented with 10% fetal bovine serum (Chemie Brunschwig) and 100 μg/ml Pen/Strep Glutamine (Thermo Fisher), DMEM/FBS/PS for simplification, and incubated at 37 °C in a humidified incubator with 5% CO_2_. mCCD_cl1_ cells (59) were maintained at 37 °C and 5% CO_2_ in DMEM/F12 (Thermo Fisher) supplemented with 5 μg/ml insulin (Sigma), 50 nM dexamethasone (Sigma), 60 nM selenium (Sigma), 5 μg/ml transferrin (Sigma), 1 nM triiodothyronine (Sigma), 5 ng/ml mouse EGF (Sigma), 100 μg/ml Pen/Strep Glutamine (Thermo Fisher), and 2% decomplemented fetal bovine serum (Thermo Fisher). MCF-7, HEK293T, and HeLa cells were transfected using Roti-Fect (ROTH), according to manufacturer’s instruction. Prior to transfection, cells were seeded on 24-well plates at a density of 5.2 × 10^4^ cells (for MCF-7 and HEK293T) or 2.6 × 10^4^ cells (for HeLa) per cm^2^ of the dish surface area. For calcium phosphate precipitation method of DNA delivery, overnight medium was aspirated and replaced with DMEM/FBS/PS containing 25 uM chloroquine. In each well, 10% (v/v) of transfection mixture was introduced to the cells, consisting of 50% (v/v) 1.2 to 1.5 μg plasmid DNA/250 mM CaCl_2_ and 50% (v/v) HBS buffer (50 mM HEPES, 280 mM NaCl, 10 mM KCl, 1.5 mM Na_2_HPO_4_, 12 mM glucose). Transfected cells were incubated at 37 °C/5% CO_2_ for 24 h before the medium was replaced with fresh DMEM/FBS/PS and continued to incubate at 37 °C/5% CO_2_ for another 24 h. Cells were harvested 48 h posttransfection. mCCD_cl1_ cells were transfected using Polyplus JetPrime according to manufacturer’s instructions. For immunocytochemistry experiments, HeLa and mCCD cells were seeded in 6-well plates on glass coverslips at 0.2 x 10^6^ cells per well. After 24 h, the cells were transfected and either left untreated or serum-starved for another 24 h.

### Luciferase reporter gene assays

Fifty nanograms of promoter- and/or enhancer-containing firefly luciferase plasmid was cotransfected along with 1 ng of pRL-SV40 *Renilla* luciferase to control for differences in transfection efficiency and extract preparation. For the study of FOXJ1 and RFX2 activity on *ADGB* promoters, 300 ng of pcDNA3.1-nV5-FOXJ1 or pcDNA3.1-HA-RFX2 was cotransfected with promoter plasmid, the total amounts of plasmid DNA used were normalized with pcDNA3.1-nV5-HisA empty vector. Luciferase activities were determined using the Dual Luciferase Reporter Assay System (Promega) as described before (60). Reporter activities were expressed as relative firefly/*Renilla* luciferase activities. All reporter gene assays were performed at least three times independently.

### dCas9-VPR-mediated activation of endogenous *ADGB* promoter

Nuclease-null-Cas9 with tandem fusion of VP64-p65-Rta tripartite activator (dCas9-VPR, Addgene #63798) (24) was delivered along with gRNAs as described before (61) to activate *ADGB* promoter activity. gRNAs candidates targeting between −1 and −1700 bp upstream of *ADGB* TSS were cloned into, and expressed from, pSPgRNA plasmid (Addgene #47108), which was a generous gift from Prof. Charles Gersbach (62) (Table S2). Prior to cloning, phosphorylated oligo duplexes were generated by incubating 0.5 μM of synthesized complementary oligo strands (5'–3') in T4 DNA ligase buffer (ThermoScientific) (40 mM Tris-HCl, 10 mM MgCl_2_, 10 mM DTT, 500 μM ATP) with T4 polynucleotide kinase (ThermoScientific) at 37 °C for 1 h, followed by heating to 95 °C for 5 min and slow cooling at the rate of −5 °C min^−1^ to 10 °C. Oligo duplexes with sticky ends complementary to *Bbs*I-digested pSPgRNA vector were then ligated into the vector. dCas9-VPR and gRNAs were delivered to HEK293T cells with Roti-Fect (ROTH) according to the manufacturer’s instruction. Cells were transfected in 24-well plates, seeded with 5.2 x 10^4^ cells per cm^2^ surface area, 24 h prior to transfection. In each well, 375 ng of dCas9-VPR was delivered together with 125 ng of gRNA(s) in antibiotic-free DMEM medium. For the transfection of gRNAs in combinations, the total amount of gRNAs was equally distributed to a total of 125 ng. Cells were incubated for 24 h before the transfection medium was replaced with fresh DMEM/FBS/PS and allowed to grow for another 24 h before RNA extraction for analysis. For dCas9-VPR activation of endogenous ADGB and subsequent immunoblot analysis, HEK293T cells were seeded on 6-well plates at a density of 5.2 × 10^4^ cells per cm^2^ of the dish surface area and were allowed to grow at 37 °C/5% CO_2_ for 24 h. For transfection, 2250 ng of dCas9-VPR and 750 ng of gRNAs mix (equal amount of gRNA AP-1 and gRNA AP-2) were delivered to HEK293T cells with Roti-Fect (ROTH), and cells were incubated for 24 h before the transfection medium was replaced with fresh DMEM/FBS/PS and allowed to grow for another 48 h.

### dCas9-mediated interference of FOXJ1 binding

Nuclease-null-Cas9 was cloned by amplifying the dCas9 gene using primer pairs with *Kpn*I and *Not*I overhangs listed in Table S2. The amplicon was subsequently digested with restriction enzymes as designed on the primers and ligated into a linearized pcDNA3 vector to generate pcDNA3-dCas9. Candidate gRNAs targeting the *Cons2* region within the *ADGB* promoter and control gRNAs targeting more distal regions (Table S2) were cloned into and expressed from a pSPgRNA plasmid (Addgene #47108) as described above. For dCas9-mediated interference of FOXJ1 binding, 800 ng of pFLAG-FOXJ1 was cotransfected with 1000 ng of pcDNA3-dCas9 and 600 ng of gRNA into HEK293T of MCF-7 cells with calcium phosphate precipitation and Roti-Fect (ROTH), respectively. Cells were incubated for 24 h before the transfection medium was replaced with fresh DMEM/FBS/PS, and allowed to grow for another 24 h.

### Chromatin immunoprecipitation

ChIP was carried out as described before with some modifications (63, 64). Briefly, cells were cross-linked by adding 1% (w/v) formaldehyde and incubated for 20 min at RT with gentle shaking. Cell fixation was interrupted by adding 110 mM glycine. Cells were scraped off and resuspended in lysis buffer following the iDeal ChIP-qPCR kit protocol (Diagenode, Liège, Belgium). To obtain genomic DNA fragments between 500 and 100 bp, cell lysates were sonicated for four rounds of ten cycles (30 s ON/30 s OFF) using the Bioruptor Pico (Diagenode) at high power setting. For immunoprecipitations, the following antibodies were used: 1 μg of rabbit polyclonal anti-IgG (C15410206, Diagenode) as negative control IP; 1 μg of mouse monoclonal anti-FOXJ1 (14-9965-82, Thermo Fisher Scientific) and 3.8 μg of mouse monoclonal anti-FLAG (F1804, Sigma Aldrich). Chromatin–antibody complexes were immunoprecipitated by DiaMag Protein A-coated magnetic beads (Diagenode). DNA isolation and de-cross-linking was carried out as described by the iDeal ChIP-qPCR kit protocol (Diagenode). Coprecipitated DNA was quantified by real-time qPCR using the primers listed in Table S2.

### Immunocytochemistry and cilia counting

The cells were washed with phosphate-buffered saline (PBS) and fixed for 10 min in 4% para-formaldehyde (PFA), followed by 3 × 5 min PBS washes, permeabilization in PBS/Triton X-100 0.2% for 10 min, 3 × 5 min PBS washes, and blocking in PBS/BSA 1% for 1 h. The cells were then incubated overnight with mouse anti-acetylated tubulin (Santa Cruz) diluted 1/500 in PBS/BSA 0.1%. On the following day, cells were washed 3 x 5 min with PBS and incubated for 1 h with secondary Alexa Fluor goat anti-mouse IgG (Invitrogen) diluted 1/300 in PBS. The slides were mounted with Fluoromount mounting medium containing DAPI (Southern Biotech) and visualized on a Nikon Eclipse fluorescent microscope (Nikon Corporation). Cilia counting was performed using ImageJ software.

### Immunohistochemistry of bovine tissue

After 20 h of fixation, the tissue was washed twice in PBS and cryoprotected in 20% saccharose solution. To avoid desiccation, the tissue was enveloped in parafilm and stored at −80 °C until further use. The samples were embedded in Neg-50 Frozen Section Medium (Thermo Scientific) and sectioned in a cryostat at −20 °C. Before immunostaining, heat-induced epitope retrieval was performed for 30 min at pH 6. Slides were permeabilized (PBS with 0.1% Triton X), blocked in blocking buffer (10% Horse serum in PBS with 0.1% Triton X), and probed with primary rabbit anti-ADGB antibody (1/100) (Sigma-Aldrich, HPA036340) in blocking buffer overnight. Incubation with secondary goat anti-rabbit CF 488 antibody (1/250) (Sigma-Aldrich, SAB4600036) or goat anti-rabbit Alkaline Phosphatase (1/500) (Sigma-Aldrich, A3687) was performed for 1 h. Fluorescent samples were counterstained with DAPI (Roche) and embedded in RotiMount Fluorcare antifading solution (Roth). For colorimetric staining, slides were incubated in NBT-BCIP (Roche) under exclusion of oxygen. Image acquisition was done either with a Leica SP5 or a fluorescence microscope BX61 (Olympus).

### Protein extraction and immunoblotting

Protein extraction and immunoblotting were performed as before with some modifications (65). Cells were lysed in NP-40 lysis buffer (10 mM Tris, pH 8.0, 1 mM EDTA, 400 mM NaCl, 0.1% NP-40, 2 μg ml^−1^ leupeptin, 2 μg ml^−1^ pepstatin, 2 μg ml^−1^ aprotinin, 1 mM PMSF) or in Triton X-100 buffer (50 mM Tris-HCl pH 7.4, 150 mM NaCl, 1% Triton X-100, 2 μg/ml aprotinin, 4 μg/ml leupeptin, 2 μg/ml pepstatin and 1 mM PMSF), and centrifuged for 10 min at 14,000 rpm at 4 °C. For the detection of CRISPRa-induced ADGB, total cell lysate was first immunoprecipitated with 0.2 μg of ADGB antibody (Sigma-Aldrich) and 25 μl bed volume of Protein-G-Sepharose beads (Sigma-Aldrich). Protein quantification was performed by Bradford assay. In total, 50 to 100 μg proteins was separated on 10% SDS acrylamide gels and electrically transferred onto nitrocellulose membranes (Amersham Protran Western blotting membranes). Following transfer membranes were blocked for 1 h with 5% dried milk/TBS-tween at room temperature and incubated overnight in 1% dried milk/TBS-tween with primary rabbit anti-ADGB (1/500) (Sigma-Aldrich, HPA036340), mouse anti-FOXJ1 (1/300) (Invitrogen, 14-9965-82), mouse anti-HA (1/100) (Santa Cruz, sc-57592), mouse anti FLAG (1/1000) (Sigma-Aldrich, F3165), or rabbit anti-CAS9 (1/2500) (ABclonal, A14997) antibodies. Membranes were then incubated for 1 h with HRP-conjugated goat anti-rabbit secondary antibody (GE Healthcare), and the signal was revealed using ECL Prime (Amersham) on a C-DiGit western blot scanner (LI-COR Biosciences) or fluorescent signals were visualized *via* a Li-COR Odyssey infrared imaging system.

### Data analysis

Results are shown as mean values ± SEM of at least three independent experiments. Statistical analysis was performed applying two-tailed or paired Student’s *t*-test or one-way analysis of variance (ANOVA) for multiple comparisons, using GraphPad Prism Version 7.0 (GraphPad Software).

## Data availability

All data are contained within the article.

## Conflict of interest

The authors declare no conflicts of interest in regard to this article.
