# Supplementary material for: Androglobin gene expression patterns and FOXJ1-dependent regulation indicate its functional association with ciliogenesis
Source: J Biol Chem. 2021 Jan 13;296:100291. doi: 10.1016/j.jbc.2021.100291 (PMC7949040; doi:10.1016/j.jbc.2021.100291)
Supplement: Supplemental Figures and Tables [file mmc1.pdf]

***Androglobin* gene expression patterns and FOXJ1-dependent regulation indicate its functional association with ciliogenesis**

Teng Wei Koay[1 *], Carina Osterhof[2 *], Ilaria M.C. Orlando[1], Anna Keppner[1], Daniel Andre[2], Schayan Yousefian[2], María Suárez Alonso[1], Miguel Correia[1], Robert Markworth[3], Johannes Schödel[4], Thomas Hankeln[2 #], David Hoogewijs[1 # §]

Supporting information

Supplemental Table S1

Publicly available transcriptome raw data used in this study

Supplemental Table S2

Primers and oligonucleotides used in this study

Supplemental Figure 1: RNA-Seq analysis on ADGB mRNA expression in human endometria subdivided into epithelial and stromal fraction on two different time points of the menstrual cycle. ADGB is predominantly expressed in the epithelium, whereas the time point during menstrual cycle has no effect on ADGB mRNA expression. Data set adapted from (8). *, $P < 0.05$; **, $P < 0.01$.

Supplemental Figure 2: Antibody staining of Adgb in bovine testes (A-D), endometrium (E) and oviduct (F).
(**A**) DAPI staining of DNA. (**B**) Fluorescent staining with anti-Adgb antibody. (**C**) Overlay of DAPI and anti-Adgb antibody staining (**D**) Control with secondary antibody only. L = Lumen; ESpg = early Spermatogonia; RSpt = round Spermatids; MSp = mature Sperm. Adgb mRNA is predominantly expressed in round Spermatids. (**E-F**) Chromogenic staining of bovine endometrium and oviduct tissue with anti-Adgb antibody. Ct = connective tissue; Ep = epithelium; Gl = gland; Sc = secretory cell; Mcc = multi-ciliated cell. Endometria show Adgb mRNA expression in epithelia and glands, whereas there is no expression in stromal cells. In oviducts, Adgb mRNA expression is restricted to multi-ciliated cells of the epithelia and no staining can be found in secretory cells.

Supplemental Figure 3: Single-cell RNA-Seq analysis of murine testis tissue.
Shown are marker genes for the different stages of spermatogenesis: Itgb1 (Spermatogonia); Prss44 (Spermatocytes); Foxj1 (elongating spermatids); Dyrk4 (elongated spermatids); Cdkn1c (mature sperm). Adgb mRNA expression strongly overlaps with expression of Foxj1 and thus correlates with later stages of spermatogenesis, as described previously (5). Data set adapted from (13).

Supplemental Figure 4: Reclustering of the subset of ciliated cells from mouse lung single-cell RNA-Seq data.
(**A**) UMAP representation of new sub-clusters (0,1,2,3) within the ciliated cells. Positivity of Adgb, Foxj1, Cdhr3 and Aqp3 expression does not correlate with any of the new clusters. (**B**) Comparison of expression of Adgb, Foxj1 and Aqp3 within the sub-clusters. Sub-clusters do not differ much in Adgb or FoxJ1 expression, except for cluster 4, which shows higher expression of Aqp3 instead. Thus, Adgb expression is rather associated with fully differentiated ciliated cells than precursors derived from the basal cell population.

Supplemental Figure 5. ***ADGB*** **promoter locus.**
UCSC Genome Browser output (*hg19*) of the *ADGB* genomic upstream region. The ENCODE integrated regulation track containing the H3K4Me3 histone mark, DNaseI hypersensitivity clusters and transcription factor ChIP-seq data are displayed. Chromatin State Segmentation by Hidden Markov models (HMM) and additional ENCODE ChIP-seq based H3K4me3 promoter histone marks from the Broad Institute, University of Washington and Stanford/Yale/USC/Harvard (SYUH) datasets are shown.

Supplemental Figure 6. Various transcription factors induce ***ADGB*** **promoter-driven luciferase activity**
(**A**) Empty control or indicated plasmids encoding different transcription factors were co-transfected with the longest *ADGB* promoter construct (pGL3-AP2014) and a *Renilla* luciferase plasmid in HeLa cells. (**B**) Empty control or increasing amounts of pcDNA3.1-GATA-3 vector were co-transfected with

the longest *ADGB* promoter construct (pGL3-AP2014) and a *Renilla* luciferase plasmid in HeLa cells. Data represent mean ± S.E.M (error bars); *, P < 0.05; **, P < 0.01; ***, P < 0.001; ****, P < 0.0001.

Supplemental Figure 7. ReMap-based *ADGB* locus.
UCSC Genome Browser output (*hg38*) of the *ADGB* locus, including the ReMap integrative dataset of transcriptional regulator ChIP-sequencing experiments in various cell lines. The 7 *ADGB* enhancers are highlighted. The blocks in the ReMap track represent transcription factors across multiple cell lines.

Supplemental Figure 8. ENCODE-based *ADGB* locus.
UCSC Genome Browser output (*hg19*) of the *ADGB* locus, including active enhancer histone marks (H3K4me1, H3K4me2, H3K27ac and H3K9ac) from ENCODE in various cell lines. Chromatin State Segmentation by Hidden Markov models (HMM) and ChIP-sequencing based histone modifications from the Broad Institute and Stanford/Yale/USC/Harvard (SYUH) are displayed.

Supplemental Figure 9. Chromatin immunoprecipitation in non-transfected cells displays absence of FOXJ1 binding. The amount of co-precipitated chromatin derived from the proximal *ADGB* promoter region using 2 primer pairs (covering +21 to -184 and -184 to -309 upstream of the *ADGB* TSS, selected based on the reporter gene assays), its upstream (5') and downstream (3') regions as well as an independent region on chromosome 7 in the *EPO* locus (56), was determined by qPCR. Data represent mean ± S.E.M (error bars).

Supplemental Figure 10. FOXJ1-mediated increase in luciferase activity from a 60 bp *ADGB* promoter fragment is significantly reduced upon mutation of *Cons1* and fully abolished upon mutation of *Cons2*
Substitution-based mutation at -96 to -92 bp (Mut1), -57 to -52 bp (Mut3), and -51 to -46 bp (Mut4) results in total loss of FOXJ1-dependent increase in *ADGB* promoter activity. The mutation at -73 to -68 bp (Mut2) reduced, but did not fully abolish FOXJ1-mediated activation (n=3 independent experiments). Data represent mean ± S.E.M (error bars); *, P < 0.05; **, P < 0.01.

Supplemental Figure 11. FOXJ1 and RFX2 transcription factors induce endogenous ADGB transcription using multiple primer pairs covering the whole *ADGB* locus.
(**A**) HEK293T cells were transiently transfected with a FOXJ1 expressing plasmid and ADGB mRNA levels were measured with RT-qPCR using primers targeting different regions of the full-length ADGB in order to include most potential splice variants. Overexpression of FOXJ1 in HEK293T cells results in increased expression of ADGB mRNA with all of the primer pairs used (n=6 independent experiments). (**B**) Similarly, the overexpression of RFX2 in HEK293T cells results in increased ADGB mRNA levels (n=3 independent experiments). ADGB expression levels were normalized to actin and displayed as relative values to cDNA of HEK293T cells transfected with equal amount of empty vector. (**C**) Schematic representation of primer pair localization. Data represent mean ± S.E.M (error bars). *, P < 0.05; **, P < 0.01. ***, P < 0.001; ****, P < 0.001.

Supplemental Figure 12. RFX2 has no direct effect on *ADGB* promoter- and enhancer-driven luciferase constructs
(**A**) SV40 promoter-driven luciferase assays coupled with upstream *ADGB* enhancer elements (Int35-AE1, Int35-AE2, 3'-AE1, 3'-AE2, Int29-AE, Int1-AE, Int12-AE) show no RFX2-dependent increase in promoter activity (n=3 independent experiments). (**B**) Reporter gene assays with various *ADGB* enhancer-dependent *ADGB* promoter-driven luciferase constructs display no RFX2-dependent regulation of *ADGB* promoter activity. Data represent mean ± S.E.M (error bars). (**C**) Immunoblotting analysis using an HA-antibody to control for RFX2 overexpression. Two unspecific bands indicate equal loading.

Supplemental file 1

Associated gene list and the full list of enriched terms of Table 1

Supplemental file 2

Associated gene list and the full list of enriched terms of Table 2

Supplemental Table S1 Publicly available transcriptome raw data used in this study

Study	Type	Organism	Tissue	Publication
PRJNA263600	bulk RNA sequencing	*B. taurus*	several organs	-
PRJEB6971	bulk RNA sequencing	*H. sapiens*	several organs	Uhlen et al. 2015
PRJNA382259	bulk RNA sequencing	*H. sapiens*	Endometria	Altmäe et al. 2017
PRJNA398141	bulk RNA sequencing	*H. sapiens*	Fallopian tubes	Schuster et al. 2015
PRJNA413049	single cell RNA sequencing	*M. musculus*	Testis	Lukassen et al. 2018
PRJNA401883	single cell RNA sequencing	*M. musculus*	Lung	Montoro et al. 2018
PRJNA360829	single cell RNA sequencing	*M. musculus*	Hypothalamus	Campbell et al. 2016

Supplemental Table S2 Primers and oligonucleotides used in this study

Activator sgRNA-guides

	**strand**	**Sequence (5' to 3', with PAM sequence in Bold)**
gRNA AP-1	sense	GCTCTCCGGGCGCTGGACGC **GGG**
gRNA AP-2	sense	TTGCGTCCCTCTGCAGCCAC **AGG**
gRNA 3'AE1	sense	AATGTGGAGGTCAAAAGGGC **AGG**

***ADGB* promoter-targeting sgRNA-guides**

	**strand**	**Sequence (5' to 3', with PAM sequence in Bold)**
gRNA-AP_A	sense	GACCACCTGAGCCCATGACT **TGG**
gRNA-AP_B	sense	TTGCGTCCCTCTGCAGCCAC **AGG**
gRNA-AP_C	sense	GCTCTCCGGGCGCTGGACGC **GGG**
gRNA-AP_D	sense	GGACGCGGGACGCCGTCTCC **TGG**
gRNA-AP_E	sense	GTCTCCTGGCAACGCAGACG **CGG**
gRNA-AP_F	anti-sense	GCGTCTGCGTTGCCAGGAGA **CGG**

Primers for RT-qPCR

**Target**	**Organism**	**Primer**	**Sequence (5' to 3')**
ADGB_A	*H. sapiens*	*forward*	TCGAAAGATTTCTATCCTTTTGGCA
		reverse	TTTGCACCTTTGCCTGCATC
ADGB_B	*H. sapiens*	*forward*	CCTGGAAACGTCCACAAGAT
		reverse	GAGGAGGTTCCCCTGAAGTC
ADGB_C	*H. sapiens*	*forward*	ACTTCAGGGGAACCTCCTCTT
		reverse	AAGCATTGGCCACAGTTCAAA
ADGB_D	*H. sapiens*	*forward*	ACGCATACTCCCCAGTAGGA
		reverse	TCCGTAAATCGGCAGCTCTC
ADGB_E	*H. sapiens*	*forward*	TTCTGCTGAGGAAGTAGCAGC
		reverse	TTGTGTCTGGTATTCTGGCTT
ADGB_F	*H. sapiens*	*forward*	AGCTGCTCAGGGAATTCAGA
		reverse	GCTTTCAGTGAGAGGCCAAC
ADGB_G	*H. sapiens*	*forward*	TTGGTCACTGAACACAATGAATCAG
		reverse	ATTTGTTTCTGTACTCTTCCCGGAT
ADGB	*H. sapiens*	*forward*	CGGAAGGAAAACATTCAAACAGG
		reverse	CGAAACTGATGAATTTCTTCCGC
ADGB	*B. taurus*	*forward*	GAGAAATGCGAGACTCCTTAGAC
		reverse	CTCCACTCTGCTTGCGGCTT
beta-actin	*H. sapiens*	*forward*	CTGGAACGGTGAAGGTGACA
		reverse	AAGGGACTTCCTGTAACAATGCA
FOXJ1	*H. sapiens*	*forward*	TCGTATGCCACGCTCATCTG
		reverse	CGGATTGAATTCTGCCAGGT
FOXJ1	*B. taurus*	*forward*	CCCTACTCATACGCCACGCT
		reverse	GCGGATGGAATTCTGCCAAGT
DNALI1	*H. sapiens*	*forward*	GGCTCGGCTACTGAAAGTCA
		reverse	CCACTCCCTTGGGGGTAGTA
CCDC151	*H. sapiens*	*forward*	CCCTAGGAATCCGAGCTGAC
		reverse	CCTTCCGGTCACCCTCTAAC
DNAAF1	*H. sapiens*	*forward*	AACTTGATGGAACGAGAACGGA
		reverse	TCGGAAAGCACATATTCTGGTC
TEKT1	*H. sapiens*	*forward*	TGGCCAAGGTCATGGAAGAGAT
		reverse	CATCACGACACAGCTCCACG
DNAH5	*B. taurus*	*forward*	TGCGACAGGAAATAACTCGGG

		reverse	CGTGGGAGGGGCAGAAATGT
SDHA	*B. taurus*	*forward*	GAGGACTTCAAGGAGAGGGTT
		reverse	CCAGTGCTGCTAAAGGGC
Glob1	*D. melanogaster*	*forward*	GGAGCTAAGTGGAAATGCTCG
		reverse	GCGGAATGTGACTAACGGCA

Primers for cloning

**Construct**	**Primer**	**Sequence (5' to 3')**
pGL3B-AP464 (-464 to -33 bp)	*forward*	GGGGTACCCCCTCGTCACATCTTTTATGAT, *Kpn*I site underlined
	reverse	CTAGCTAGCTAGTCCGCGTCTGCGTTGCCAGGA, *Nhe*I site underlined
pGL3B-AP1064 (-1064 to -33 bp)	*forward*	GGGGTACCCCATCTCTGAAGTGAGGTTATA, *Kpn*I site underlined
	reverse	CTAGCTAGCTAGTCCGCGTCTGCGTTGCCAGGA, *Nhe*I site underlined
pGL3B-AP2014 (-2014 to -33 bp)	*forward*	GGGGTACCCCGACCCCAGGTTTTCAAGCCA, *Kpn*I site underlined
	reverse	CTAGCTAGCTAGTCCGCGTCTGCGTTGCCAGGA, *Nhe*I site underlined
pGL3B-AP2014-1065 (-2014 to -1065 bp)	*forward*	TAATAGGCAAGGTACCCTCGTCACATCTTTTATGAT, *Kpn*I site underlined
	reverse	CCTCACTTCGCTAGCATCTGACACACAGCCCTTAG, *Nhe*I site underlined
pGL3B-AP1064-465 (-1064 to -465 bp)	*forward*	GGCTGTGTGGGTACCATCTCTGAAGTGAGGTTATATTG, *Kpn*I site underlined
	reverse	TGAAAACCTGCTAGCCAGAAATTAACACAGTGGGTG, *Nhe*I site underlined
pGL3B-AP464-1 (-464 to -1 bp)	*forward*	AATTTATTAAGGTACCGACCCCAGGTTTTCAAGCCA, *Kpn*I site underlined
	reverse	ATCTATATATGCTAGCGCTCTGAGCAAAGAGCCTGC, *Nhe*I site underlined
pGL3B-AP464-271 (-464 to -271 bp)	*forward*	AATTTATTAAGGTACCGACCCCAGGTTTTCAAGCCA, *Kpn*I site underlined
	reverse	TTATAGTTATGCTAGCCCTACACTGCCTGGCTTTGC, *Nhe*I site underlined
pGL3B-AP270-141 (-270 to -141 bp)	*forward*	ATACATACAGGGTACCTCCTGCAGAACTCCAATTAATC, *Kpn*I site underlined
	reverse	TGTAGATAGAGCTAGCAGCATAAACTGAAATCCCAC, *Nhe*I site underlined
pGL3B-AP140-1 (-140 to -1 bp)	*forward*	TATTTAAATTGGTACCCTTGCGTCCCTCTGCAGCCA, *Kpn*I site underlined
	reverse	ATCTATATATGCTAGCGCTCTGAGCAAAGAGCCTGC, *Nhe*I site underlined
pGL3B-AP130-11 (-130 to -11bp)	*forward*	CCTTGGGTACCTCTGCAGCCACAGGCCGCAGAG, *Kpn*I site underlined
	reverse	CATTCGCTAGCAAGAGCCTGCGGGCGCGCTC, *Nhe*I site underlined
pGL3B-AP120-21 (-120 to -21 bp)	*forward*	CTATAGGTACCCAGGCCGCAGAGCCCGCCCCCAG, *Kpn*I site underlined
	reverse	AAAGAGCTAGCGGGCGCGCTCGGCTCCGCGTC, *Nhe*I site underlined
pGL3B-AP110-31 (-110 to -31 bp)	*forward*	ATATTGGTACCAGCCCGCCCCCAGGGCCCCG, *Kpn*I site underlined
	reverse	TGAGTGCTAGCGGCTCCGCGTCTGCGTTGC, *Nhe*I site underlined
pGL3B-AP100-41	*forward*	TATATGGTACCCAGGGCCCCGCCCCCGCTCT,

(-100 to -41 bp)		*Kpn*I site underlined
	reverse	CGGCTGCTAGCCTGCGTTGCCAGGAGACGGCGT, *Nhe*I site underlined
pGL3B-AP140-71 (-140 to -71 bp)	*forward*	TATTTAAATTGGTACCCTTGCGTCCCTCTGCAGCCA, *Kpn*I site underlined
	reverse	ATTAAGCTAGCCAGCGCCCGGAGAGCG, *Nhe*I site underlined
pGL3B-AP70-1 (-70 to -1 bp)	*forward*	TATATGGTACCGACGCGGGACGCCGTCTCC, *Kpn*I site underlined
	reverse	ATCTATATATGCTAGCGCTCTGAGCAAAGAGCCTGC, *Nhe*I site underlined
pGL3-AP(464-1)-Int35-AE1	*forward*	CGATAGGATCCAACTTCAAGAAACCTCCTTCATAG, *BamH*I site underlined
	reverse	CACTGGGATCCTTTTAAGCTGGATGTGGTGG, *BamH*I site underlined
pGL3-AP(464-1)-Int35-AE2	*forward*	CGATAGGATCCCTAAAGGAACTCTCCCAGCT, *BamH*I site underlined
	reverse	CACTGGGATCCAATGGGAGTGAACTCAAAGC, *BamH*I site underlined
pGL3-AP(464-1)-3'-AE1	*forward*	CGATAGGATCCCTCTGTTGCCCAACTGCAGT, *BamH*I site underlined
	reverse	CACTGGGATCCTGTCCAAAATTAGTCGACCA, *BamH*I site underlined
pGL3-AP(464-1)-3'-AE2	*forward*	CGATAGGATCCAACAAAAAAACACGAGTCACAG, *BamH*I site underlined
	reverse	CACTGGGATCCTCTTTTCCACCCTCCTCACA, *BamH*I site underlined
pGL3-AP(464-1)-Int29-AE	*forward*	CGATAGGATCCCAAACACTTGATGGTAGCCACC, *BamH*I site underlined
	reverse	CACTGGGATCCACGGTATGAGCTAAGAGATTTTGC, *BamH*I site underlined
pGL3-AP(464-1)-Int1-AE	*forward*	CGATAGGATCCTCAAATCTTCTGGGTCAATT, *BamH*I site underlined
	reverse	CACTGGGATCCTAAGTAGTAAAAGTCTAAAACC, *BamH*I site underlined
pGL3-AP(464-1)-Int12-AE	*forward*	CGATAGGATCCTCTTAGGAACACTGTCTTTG, *BamH*I site underlined
	reverse	CACTGGGATCCAATTCACTTGTAACTAAACG, *BamH*I site underlined
pGL3P-Int35-AE1	*forward*	CCATTTTTAAGGTACCAACTTCAAGAAACCTCCTTCATAG, *Kpn*I site underlined
	reverse	TGGGAGTATTGCTAGCTTTTAAGCTGGATGTGGTGG, *Nhe*I site underlined
pGL3P-Int35-AE2	*forward*	ATAAATGACAGGTACCCTAAAGGAACTCTCCCAGCT, *Kpn*I site underlined
	reverse	CTGTATATCAGCTAGCAATGGGAGTGAACTCAAAGC, *Nhe*I site underlined
pGL3P-3'-AE1	*forward*	TTAATACTTAGGTACCCTCTGTTGCCCAACTGCAGT, *Kpn*I site underlined
	reverse	ATGTGTAGCTGCTAGCTGTCCAAAATTAGTCGACCA, *Nhe*I site underlined
pGL3P-3'-AE2	*forward*	CCCAAGGAAAGGTACCAACAAAAAAACACGAGTCACAG, *Kpn*I site underlined
	reverse	CATAATTCAAGCTAGCTCTTTTCCACCCTCCTCACA, *Nhe*I site underlined
pGL3P-Int29-AE	*forward*	GGGGTACCCCCAAACACTTGATGGT, *Kpn*I site underlined
	reverse	TAGCTAGCTAGACGGTATGAGCTAAG, *Nhe*I site underlined

pGL3P-Int1-AE	*forward*	CAGATCTCATGGTACCTCAAATCTTCTGGGTCAATT, *Kpn*I site underlined
	reverse	GTTCCAGCGTGCTAGCTAAGTAGTAAAAGTCTAAAAC C, *Nhe*I site underlined
pGL3P-Int12-AE	*forward*	CTATCACAACGGTACCTCTTAGGAACACTGTCTTTG, *Kpn*I site underlined
	reverse	GTCAGTGATCGCTAGCAATTCACTTGTAACTAAACG, *Nhe*I site underlined
pFLAG-FOXJ1	*forward*	AGTTTGTACAGTCGACTATGGCGGAGAGCTGGCTG, *Sal*I site underlined
	reverse	TATCACCACTGGTACCTTACAAGAAGGCCCCCACGCT G, *Kpn*I site underlined
pFLAG-RFX2	*forward*	ACTACTAGATCTAATGCAGAATTCCGAGGGTGGAG, *Bgl*II site underlined
	reverse	ACTACTGTCGACCTAGATGCCCTGCAGGGAGTG, *Nhe*I site underlined
pcDNA3-dCas9	*forward*	ACCCTTGGTACCATGGACAAGAAGTACTCCATTG, *Kpn*I site underlined
	reverse	TTATTAAGAGCGGCCGCTTAGTCTCCACCGAGCTGAG AGAGGTC, *Not*I site underlined

Mutations in the *ADGB* promoter

Construct	Sequence from -41 to -100 bp (5' to 3')
pGL3B-AP140-1_Mut1 (mutation at - 96 to - 92 bp)	CAGGAAAAAGCCCCCGCTCTCCGGGCGCTGGACGCGGGACGCCG TCTCCTGGCAACGCAG, mutation site underlined
pGL3B-AP140-1_Mut2 (mutation at -73 to -68 bp)	CAGGGCCCCGCCCCCGCTCTCCGGGCGAAATTTGCGGGACGCCG TCTCCTGGCAACGCAG, mutation site underlined
pGL3B-AP140-1_Mut3 (mutation at -57 to -52 bp)	CAGGGCCCCGCCCCCGCTCTCCGGGCGCTGGACGCGGGACGCCA AAAAATGGCAACGCAG, mutation site underlined
pGL3B-AP140-1_Mut4 (mutation at -51 to -46 bp)	CAGGGCCCCGCCCCCGCTCTCCGGGCGCTGGACGCGGGACGCCG TCTCCAAATTTCGCAG, mutation site underlined

ChIP qPCR primers

Target	Primer	Sequence (5' to 3')
ADGB-184/+21	*forward*	AACTCCAAGTTCCTCCACGG
	reverse	AGCCGATCTATGTAGGGCTGA
ADGB-309/-184	*forward*	ATGTCTATGGTTCCGAAGGGC
	reverse	TGGTGTCTGGGGAGAAATCG
3’ ADGB	*forward*	TTCCTCCCATAGTTGGCTTG
	reverse	CAAGACGCGTGTGAAACTGT
5’ ADGB	*forward*	AGTGCATGTTTGAAGCCCTCT
	reverse	CAACGTGAGTGGGTGACACT
Chr. 7	*forward*	GGTGCTTGGTCAGGAGTTGA
	reverse	GACACGTTCTCGTCCAACCT

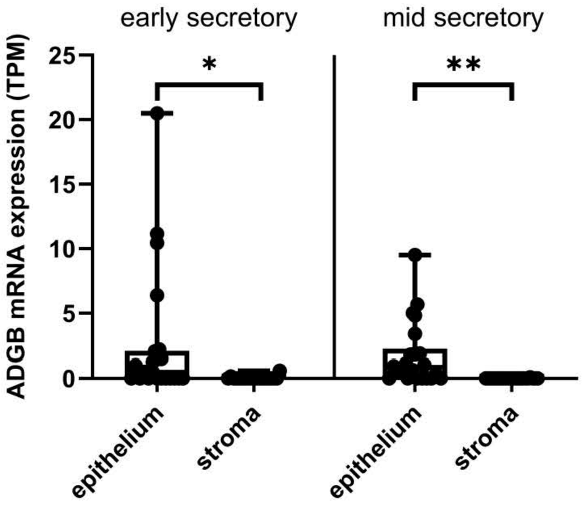

Supplemental Figure 1: RNA-Seq analysis on ADGB mRNA expression in human endometria subdivided into epithelial and stromal fraction on two different time points of the menstrual cycle. ADGB is predominantly expressed in the epithelium, whereas the time point during menstrual cycle has no effect on ADGB mRNA expression. Data set adapted from (8). *, $P < 0.05$; **, $P < 0.01$.

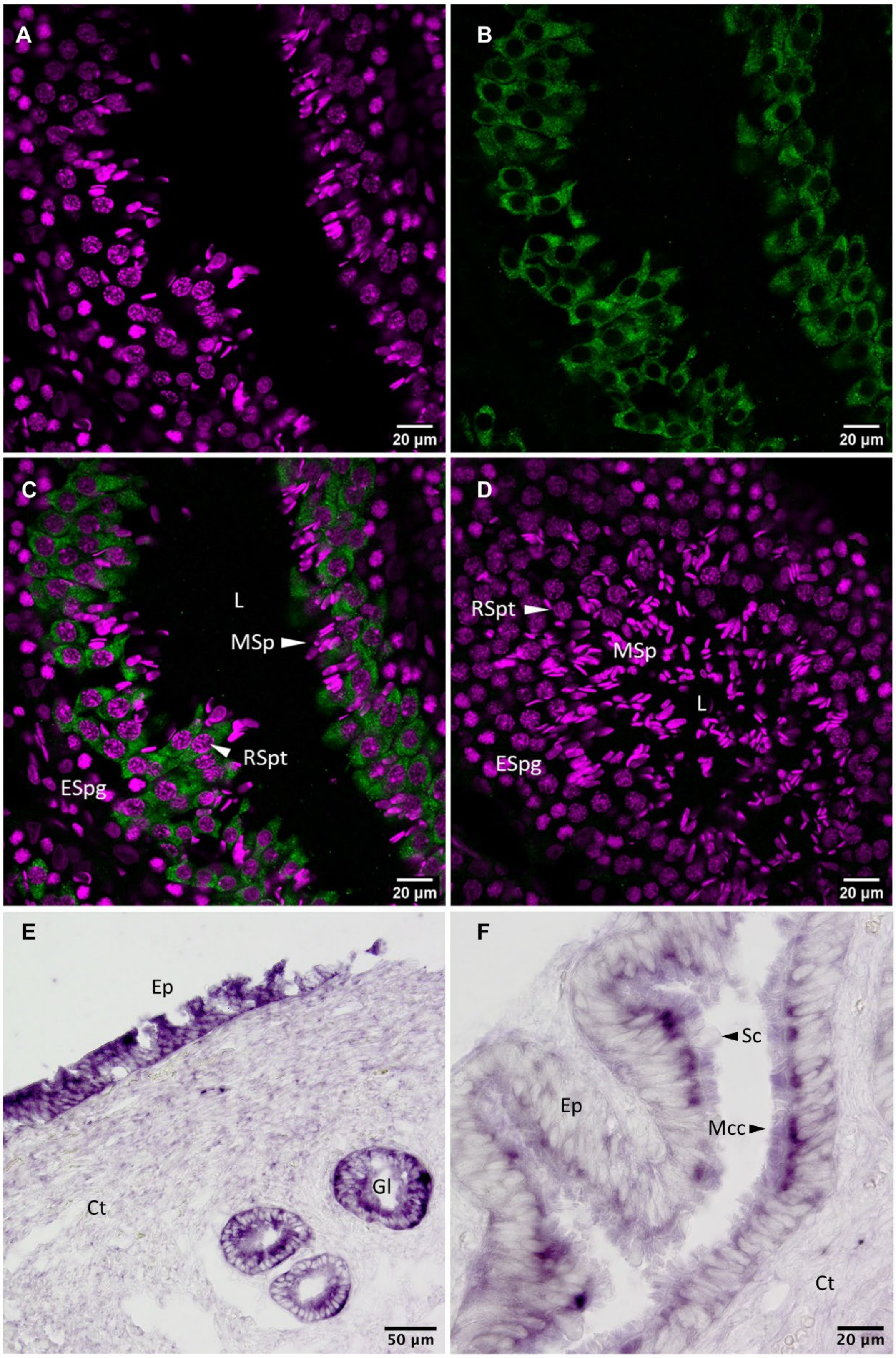

Supplemental Figure 2: Antibody staining of Adgb in bovine testes (A-D), endometrium (E) and oviduct (F).

(**A**) DAPI staining of DNA. (**B**) Fluorescent staining with anti-Adgb antibody. (**C**) Overlay of DAPI and anti-Adgb antibody staining (**D**) Control with secondary antibody only. L = Lumen; ESpg = early Spermatogonia; RSpt = round Spermatids; MSp = mature Sperm. Adgb mRNA is predominantly

expressed in round Spermatids. (**E-F**) Chromogenic staining of bovine endometrium and oviduct tissue with anti-Adgb antibody. Ct = connective tissue; Ep = epithelium; Gl = gland; Sc = secretory cell; Mcc = multi-ciliated cell. Endometria show Adgb mRNA expression in epithelia and glands, whereas there is no expression in stromal cells. In oviducts, Adgb mRNA expression is restricted to multi-ciliated cells of the epithelia and no staining can be found in secretory cells.

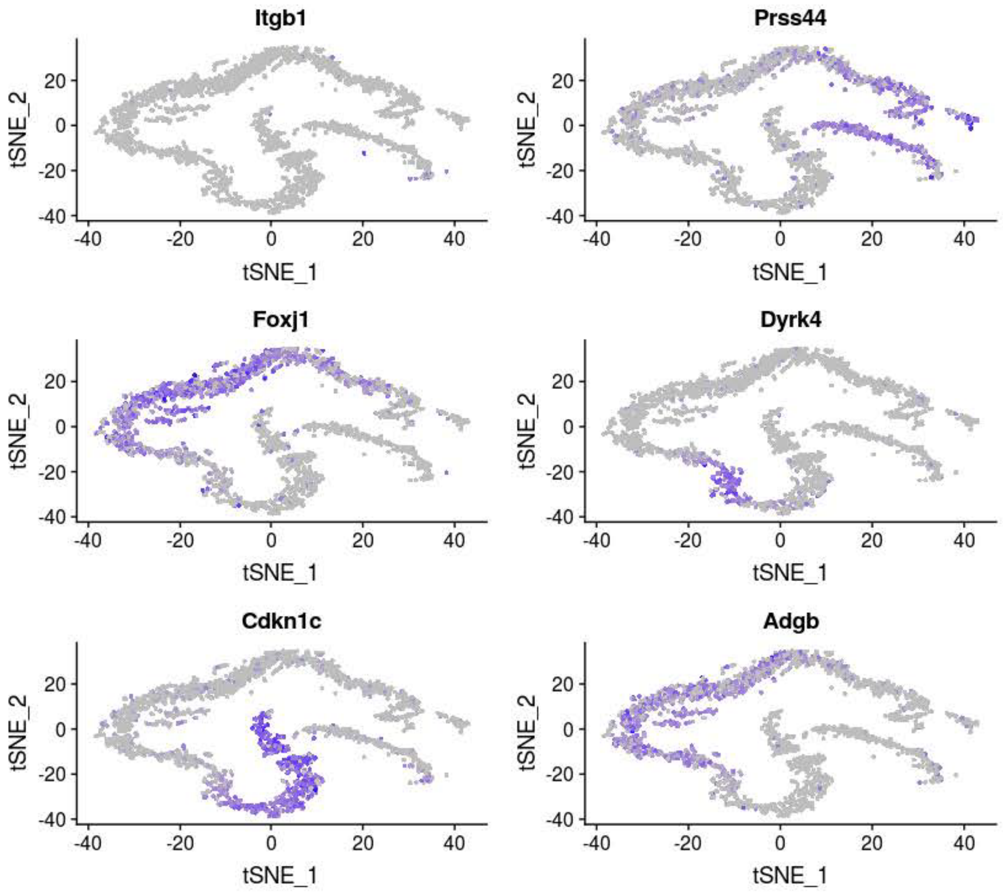

Supplemental Figure 3: Single-cell RNA-Seq analysis of murine testis tissue.
Shown are marker genes for the different stages of spermatogenesis: Itgb1 (Spermatogonia); Prss44 (Spermatocytes); Foxj1 (elongating spermatids); Dyrk4 (elongated spermatids); Cdkn1c (mature sperm). Adgb mRNA expression strongly overlaps with expression of Foxj1 and thus correlates with later stages of spermatogenesis, as described previously (5). Data set adapted from (13).

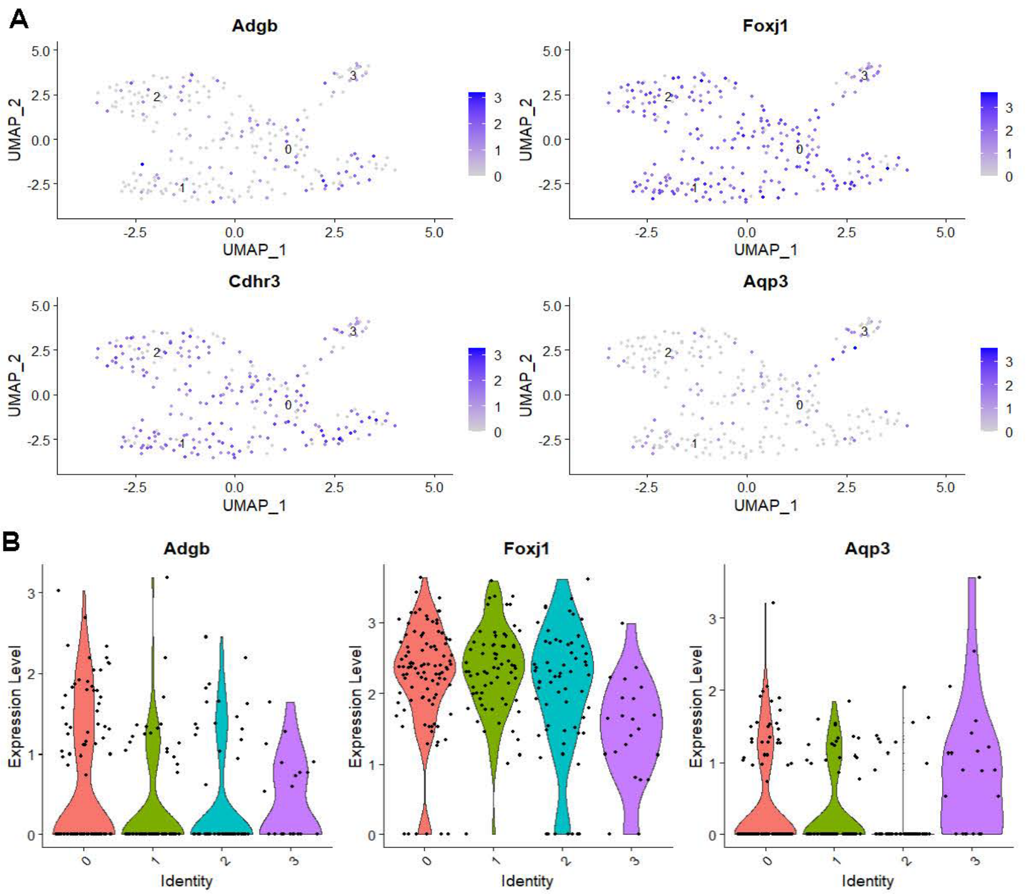

Supplemental Figure 4: Reclustering of the subset of ciliated cells from mouse lung single-cell RNA-Seq data.

(**A**) UMAP representation of new sub-clusters (0,1,2,3) within the ciliated cells. Positivity of Adgb, Foxj1, Cdhr3 and Aqp3 expression does not correlate with any of the new clusters. (**B**) Comparison of expression of Adgb, Foxj1 and Aqp3 within the sub-clusters. Sub-clusters do not differ much in Adgb or FoxJ1 expression, except for cluster 4, which shows higher expression of Aqp3 instead. Thus, Adgb expression is rather associated with fully differentiated ciliated cells than precursors derived from the basal cell population.

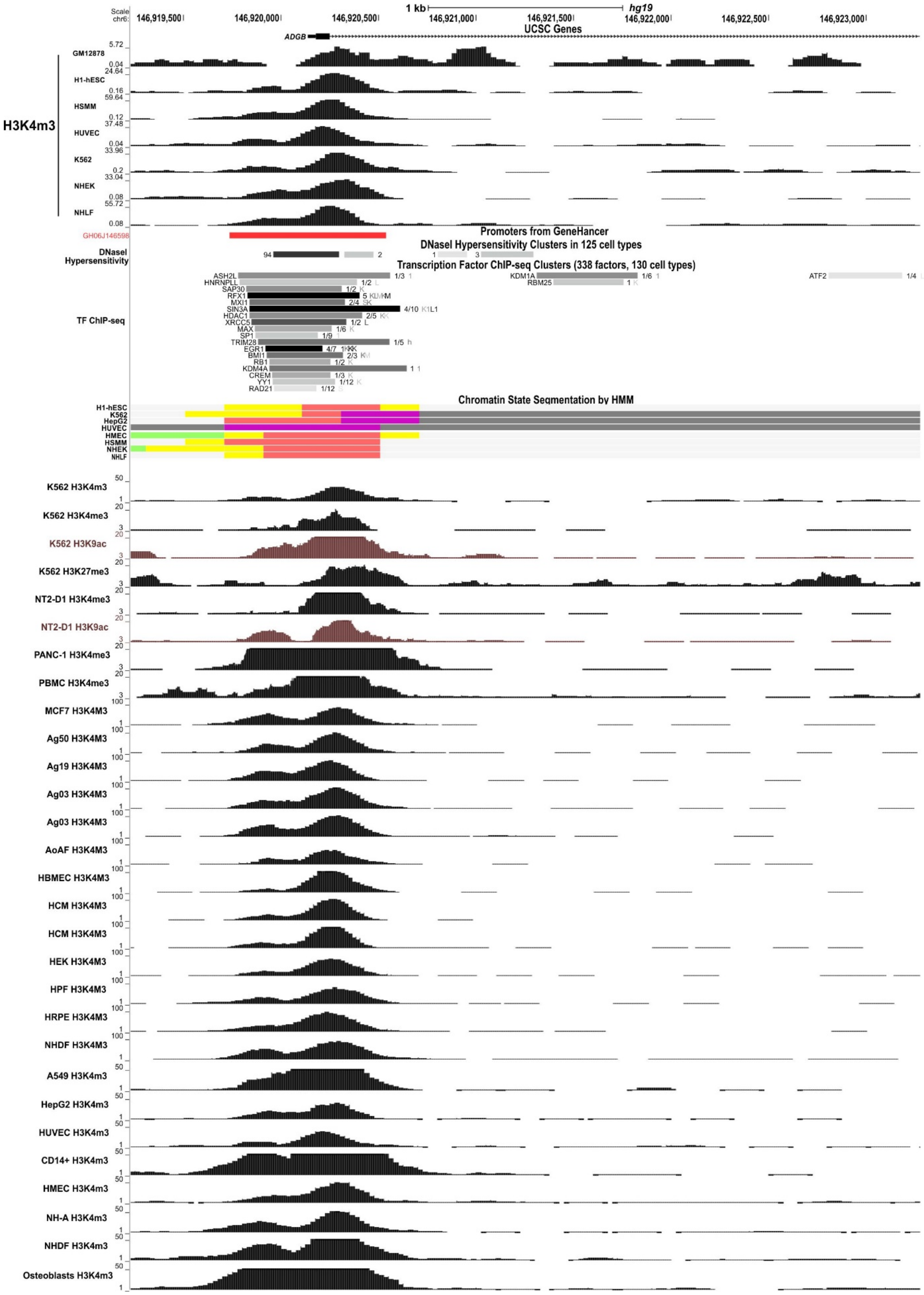

Supplemental Figure 5. ***ADGB*** **promoter locus.**

UCSC Genome Browser output (*hg19*) of the *ADGB* genomic upstream region. The ENCODE integrated regulation track containing the H3K4Me3 histone mark, DNaseI hypersensitivity clusters and transcription factor ChIP-seq data are displayed. Chromatin State Segmentation by Hidden Markov

models (HMM) and additional ENCODE ChIP-seq based H3K4me3 promoter histone marks from the Broad Institute, University of Washington and Stanford/Yale/USC/Harvard (SYUH) datasets are shown.

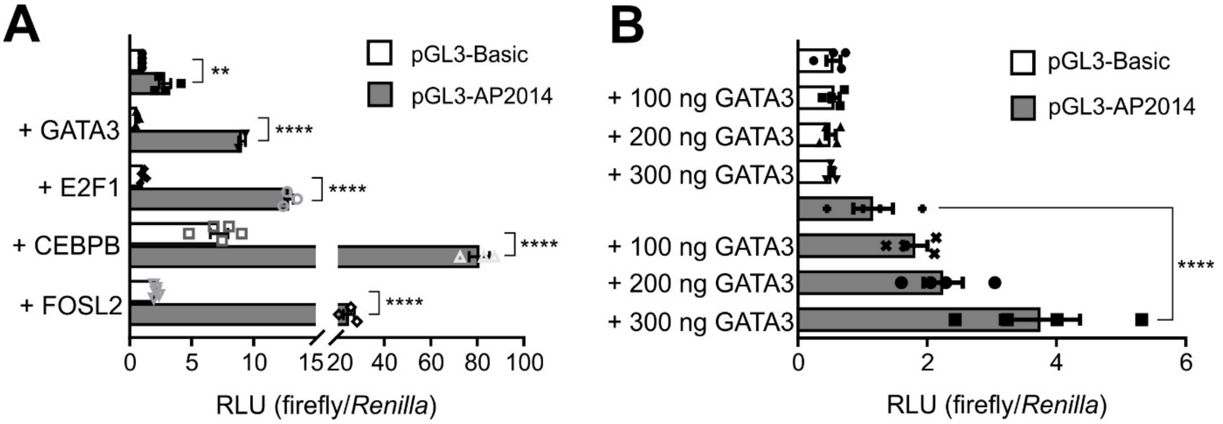

Supplemental Figure 6. Various transcription factors induce *ADGB* promoter-driven luciferase activity

(**A**) Empty control or indicated plasmids encoding different transcription factors were co-transfected with the longest *ADGB* promoter construct (pGL3-AP2014) and a *Renilla* luciferase plasmid in HeLa cells. (**B**) Empty control or increasing amounts of pcDNA3.1-GATA-3 vector were co-transfected with the longest *ADGB* promoter construct (pGL3-AP2014) and a *Renilla* luciferase plasmid in HeLa cells. Data represent mean ± S.E.M (error bars); *, $P < 0.05$; **, $P < 0.01$; ***, $P < 0.001$; ****, $P < 0.0001$.

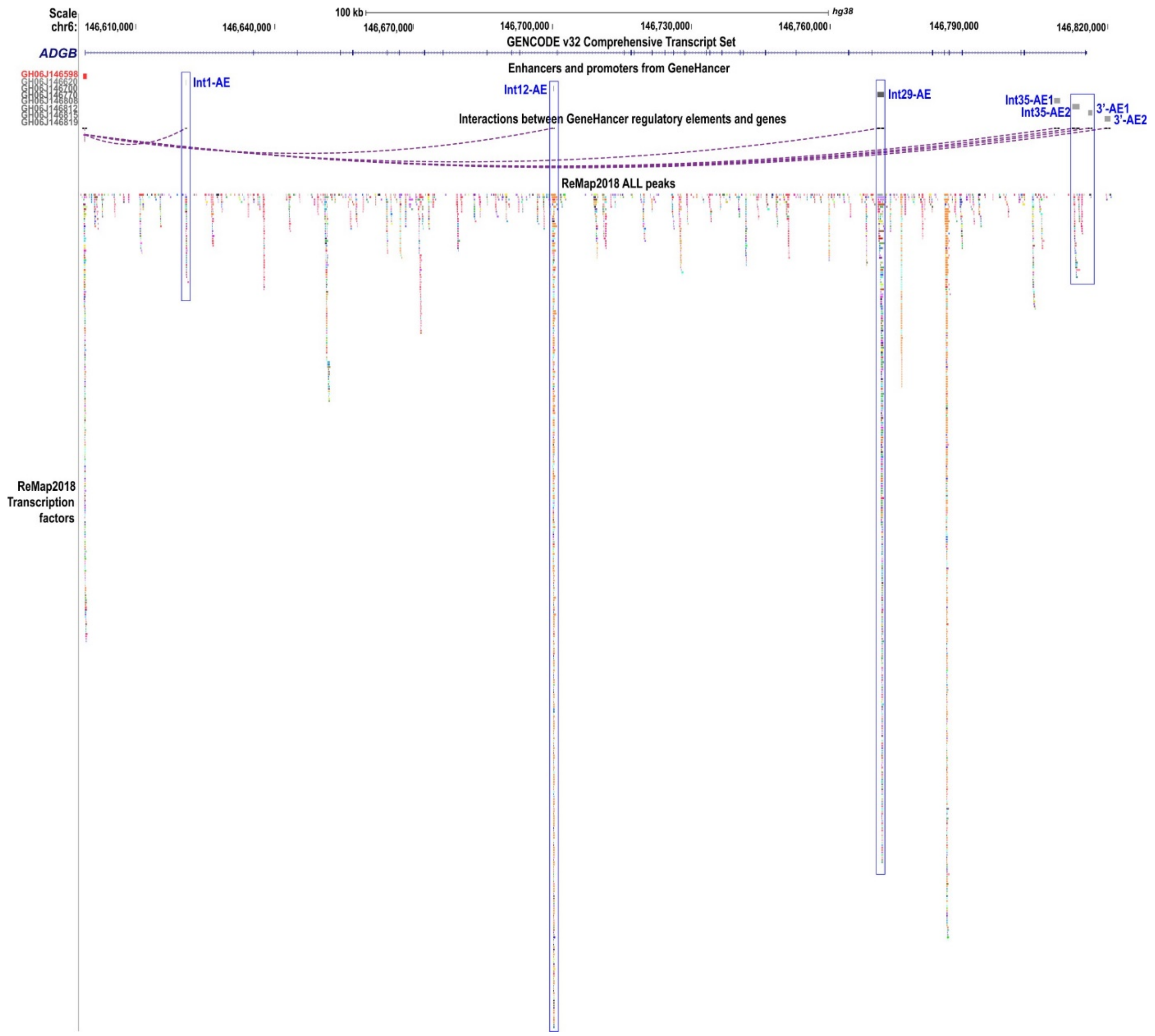

Supplemental Figure 7. ReMap-based *ADGB* locus.
UCSC Genome Browser output (*hg38*) of the *ADGB* locus, including the ReMap integrative dataset of transcriptional regulator ChIP-sequencing experiments in various cell lines. The 7 *ADGB* enhancers are highlighted. The blocks in the ReMap track represent transcription factors across multiple cell lines.

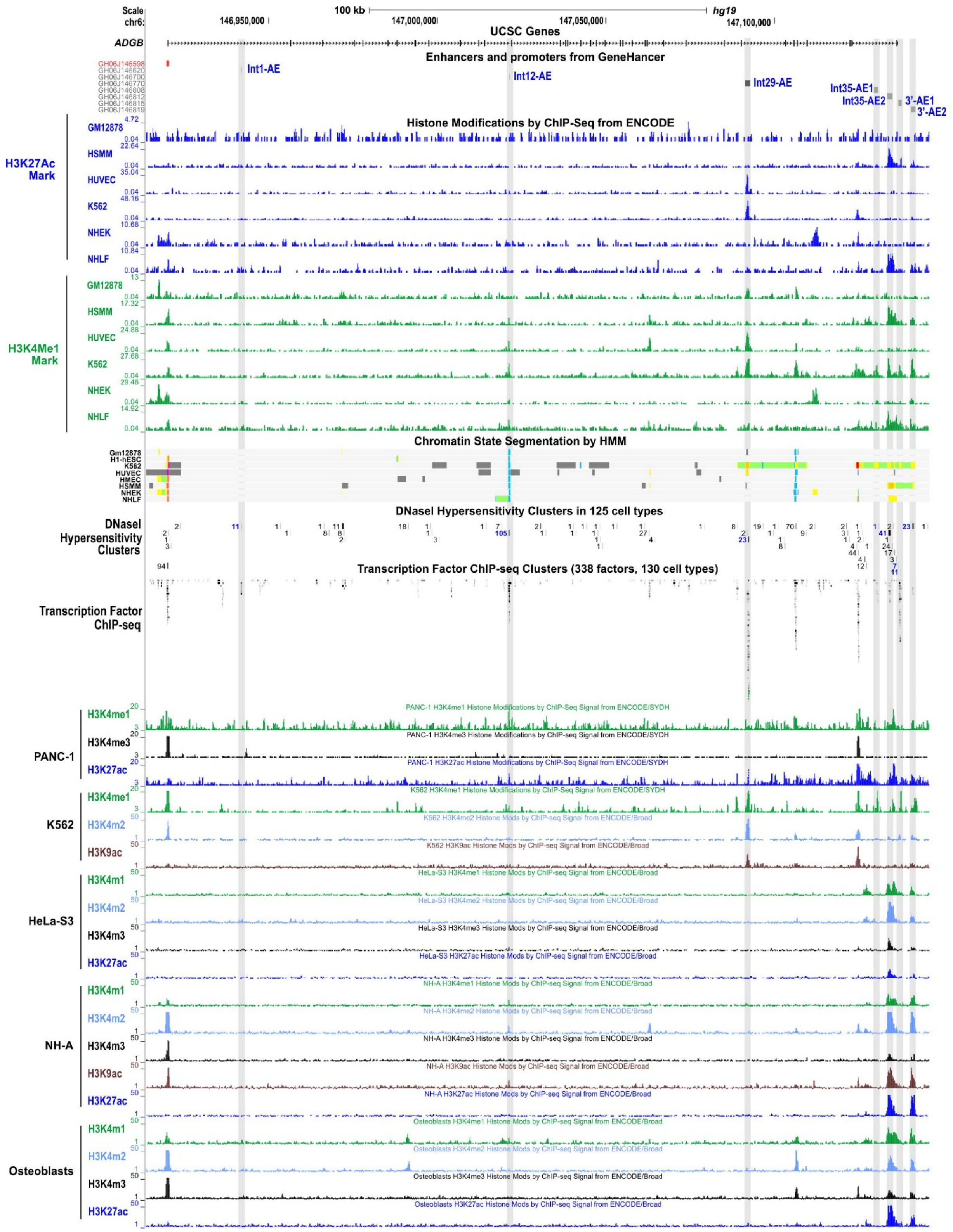

Supplemental Figure 8. ENCODE-based *ADGB* locus.

UCSC Genome Browser output (*hg19*) of the *ADGB* locus, including active enhancer histone marks (H3K4me1, H3K4me2, H3K27ac and H3K9ac) from ENCODE in various cell lines. Chromatin State Segmentation by Hidden Markov models (HMM) and ChIP-sequencing based histone modifications from the Broad Institute and Stanford/Yale/USC/Harvard (SYUH) are displayed.

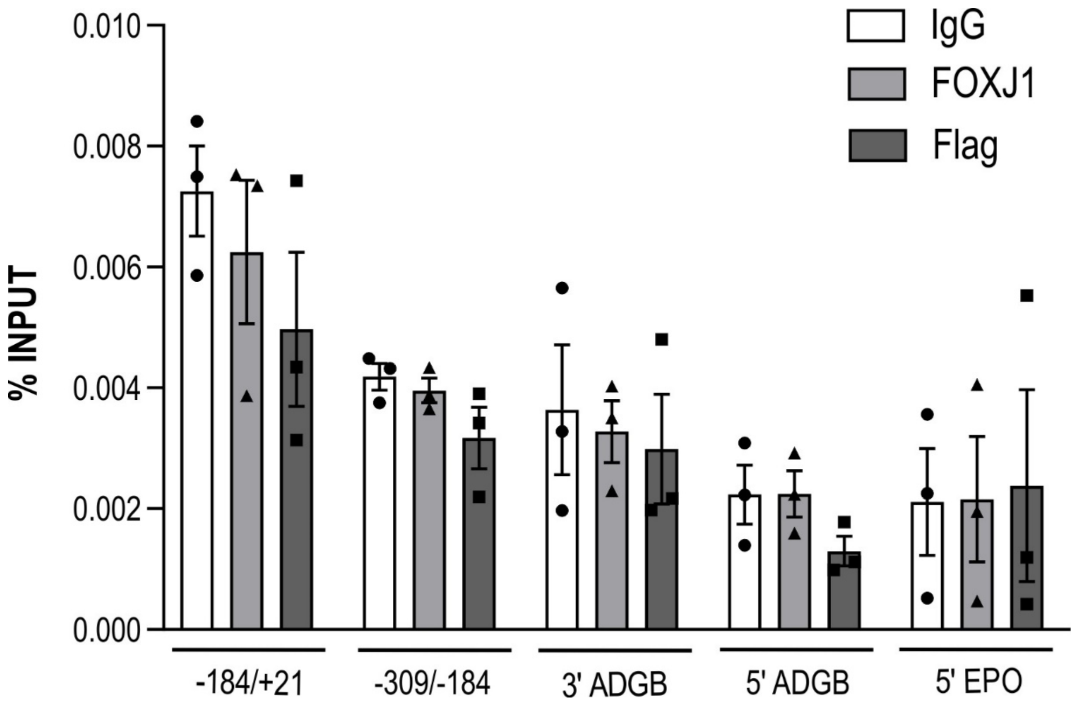

Supplemental Figure 9. Chromatin immunoprecipitation in non-transfected cells displays absence of FOXJ1 binding. The amount of co-precipitated chromatin derived from the proximal *ADGB* promoter region using 2 primer pairs (covering +21 to -184 and -184 to -309 upstream of the *ADGB* TSS, selected based on the reporter gene assays), its upstream (5') and downstream (3') regions as well as an independent region on chromosome 7 in the *EPO* locus (56), was determined by qPCR. Data represent mean ± S.E.M (error bars).

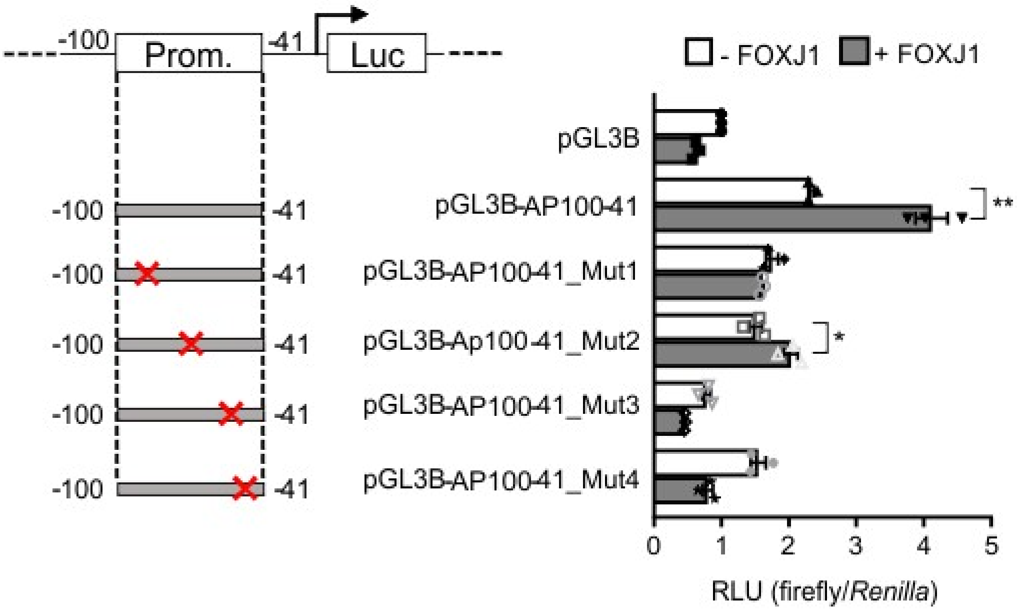

Supplemental Figure 10. FOXJ1-mediated increase in luciferase activity from a 60 bp *ADGB* promoter fragment is significantly reduced upon mutation of *Cons1* and fully abolished upon mutation of *Cons2*

Substitution-based mutation at -96 to -92 bp (Mut1), -57 to -52 bp (Mut3), and -51 to -46 bp (Mut4) results in total loss of FOXJ1-dependent increase in *ADGB* promoter activity. The mutation at -73 to -68 bp (Mut2) reduced, but did not fully abolish FOXJ1-mediated activation (n=3 independent experiments). Data represent mean ± S.E.M (error bars); *, P < 0.05; **, P < 0.01.

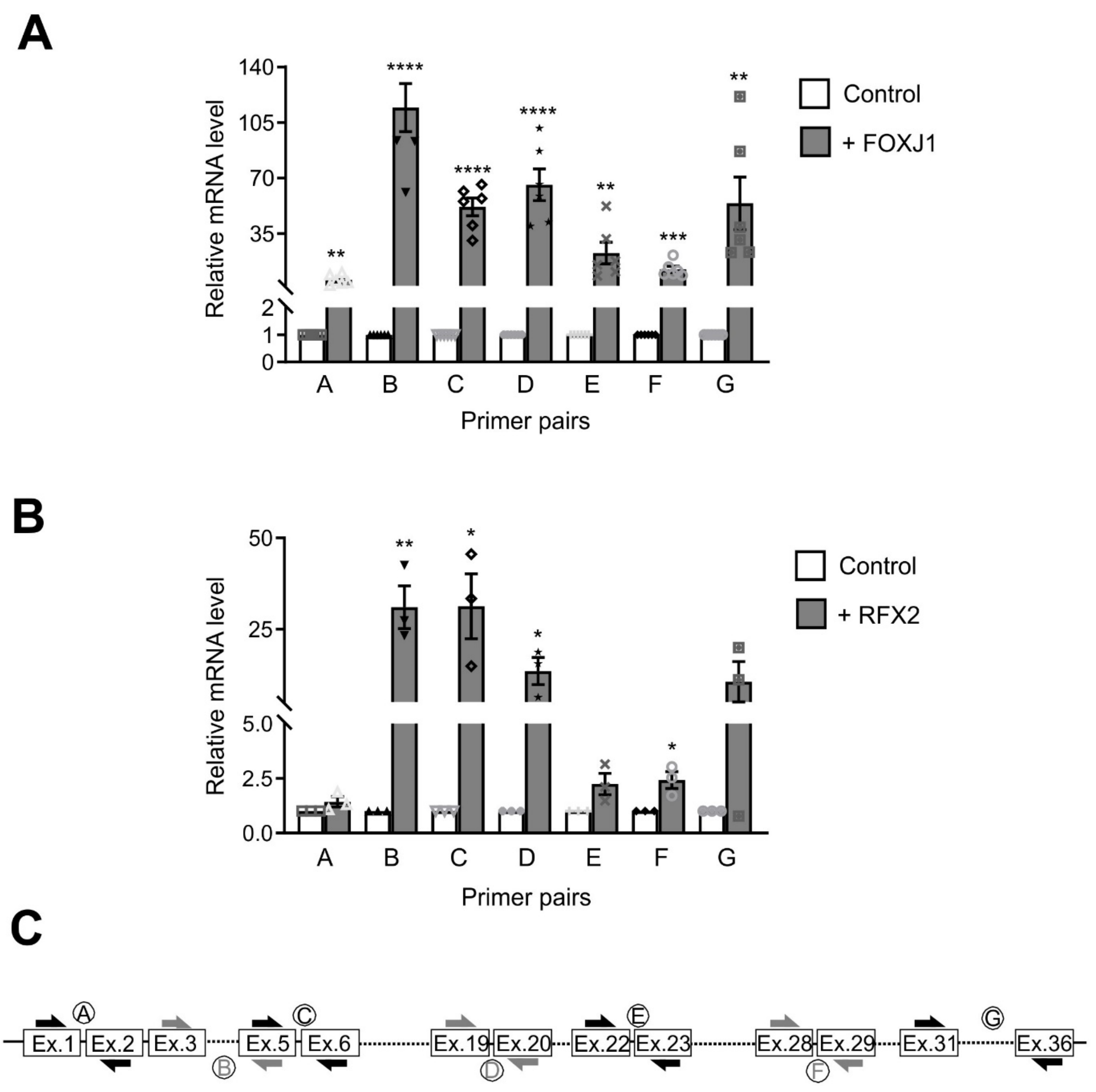

Supplemental Figure 11. FOXJ1 and RFX2 transcription factors induce endogenous ADGB transcription using multiple primer pairs covering the whole *ADGB* locus.

(**A**) HEK293T cells were transiently transfected with a FOXJ1 expressing plasmid and ADGB mRNA levels were measured with RT-qPCR using primers targeting different regions of the full-length ADGB in order to include most potential splice variants. Overexpression of FOXJ1 in HEK293T cells results in increased expression of ADGB mRNA with all of the primer pairs used (n=6 independent experiments). (**B**) Similarly, the overexpression of RFX2 in HEK293T cells results in increased ADGB mRNA levels (n=3 independent experiments). ADGB expression levels were normalized to actin and displayed as relative values to cDNA of HEK293T cells transfected with equal amount of empty vector. (**C**) Schematic representation of primer pair localization. Data represent mean ± S.E.M (error bars). *, P < 0.05; **, P < 0.01. ***, P < 0.001; ****, P < 0.001.

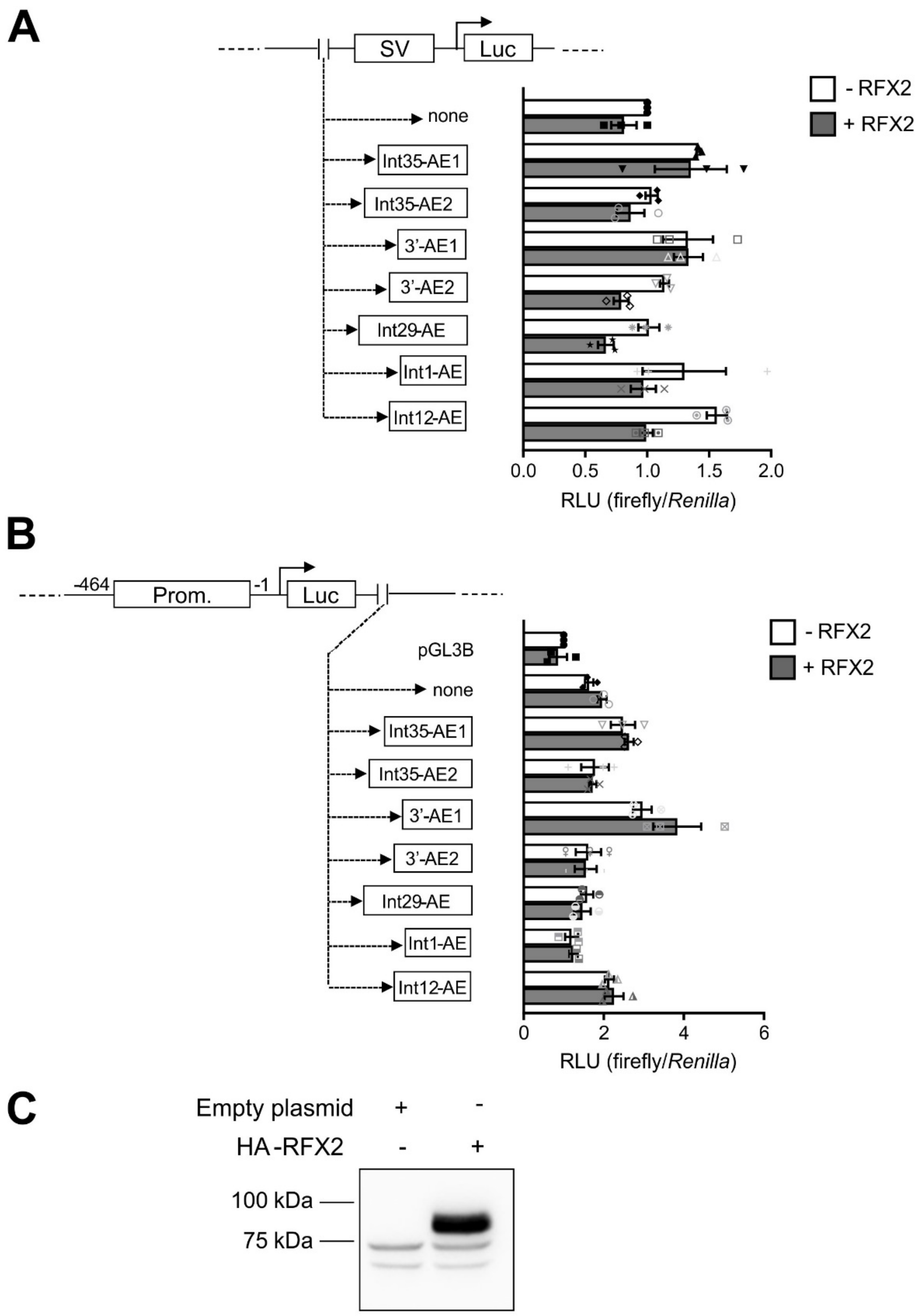

Supplemental Figure 12. RFX2 has no direct effect on *ADGB* promoter- and enhancer-driven luciferase constructs

(**A**) SV40 promoter-driven luciferase assays coupled with upstream *ADGB* enhancer elements (Int35-AE1, Int35-AE2, 3'-AE1, 3'-AE2, Int29-AE, Int1-AE, Int12-AE) show no RFX2-dependent increase in promoter activity (n=3 independent experiments). (**B**) Reporter gene assays with various *ADGB* enhancer-dependent *ADGB* promoter-driven luciferase constructs display no RFX2-dependent regulation of *ADGB* promoter activity. Data represent mean ± S.E.M (error bars). (**C**) Immunoblotting analysis using an HA-antibody to control for RFX2 overexpression. Two unspecific bands indicate equal loading.
